# Radiogenomics Consortium Genome-Wide Association Study Meta-Analysis of Late Toxicity After Prostate Cancer Radiotherapy

**DOI:** 10.1093/jnci/djz075

**Published:** 2019-05-16

**Authors:** Sarah L Kerns, Laura Fachal, Leila Dorling, Gillian C Barnett, Andrea Baran, Derick R Peterson, Michelle Hollenberg, Ke Hao, Antonio Di Narzo, Mehmet Eren Ahsen, Gaurav Pandey, Søren M Bentzen, Michelle Janelsins, Rebecca M Elliott, Paul D P Pharoah, Neil G Burnet, David P Dearnaley, Sarah L Gulliford, Emma Hall, Matthew R Sydes, Miguel E Aguado-Barrera, Antonio Gómez-Caamaño, Ana M Carballo, Paula Peleteiro, Ramón Lobato-Busto, Richard Stock, Nelson N Stone, Harry Ostrer, Nawaid Usmani, Sandeep Singhal, Hiroshi Tsuji, Takashi Imai, Shiro Saito, Rosalind Eeles, Kim DeRuyck, Matthew Parliament, Alison M Dunning, Ana Vega, Barry S Rosenstein, Catharine M L West

**Affiliations:** 1 Departments of Radiation Oncology and Surgery, University of Rochester Medical Center, Rochester, NY; 2 Department of Oncology; 3 Department of Public Health and Primary Care; 4 Centre for Cancer Genetic Epidemiology, Strangeways Research Laboratory, University of Cambridge, Cambridge, UK; Department of Oncology, Cambridge University Hospitals NHS Foundation Trust, Cambridge, UK; 5 Department of Biostatistics and Computational Biology, University of Rochester Medical Center, Rochester, NY; 6 Department of Computational Biology, University of Rochester, Rochester, NY; 7 Department of Genetics and Genomic Sciences and Icahn Institute for Data Science and Genomic Technology, Icahn School of Medicine at Mount Sinai, New York, NY; 8 Division of Biostatistics and Bioinformatics, Department of Epidemiology and Public Health, University of Maryland Greenebaum Cancer Center, School of Medicine, University of Maryland, Baltimore; 9 Division of Cancer Sciences, the University of Manchester, Manchester Academic Health Science Centre, Christie Hospital, Manchester, UK; 10 Academic Urooncology Unit, The Institute of Cancer Research and Royal Marsden NHS Foundation Trust, London, UK; 11 Clinical Trials and Statistics Unit, The Institute of Cancer Research, London, UK; 12 MRC Clinical Trials Unit at UCL, Institute of Clinical Trials and Methodology, University College London, London, UK; 13 Fundación Pública Galega de Medicina Xenómica-Servizo Galego de Saude (SERGAS & Instituto de Investigación Sanitaria de Santiago de Compostela (IDIS), Santiago de Compostela, Spain; 14 Department of Radiation Oncology; 15 Department of Medical Physics; 16 Complexo Hospitalario Universitario de Santiago, SERGAS, Santiago de Compostela, Spain; Department of Radiation Oncology; 17 Department of Urology; 18 Icahn School of Medicine at Mount Sinai, New York, NY; Departments of Pathology and Genetics, Albert Einstein College of Medicine, Bronx, NY; 19 Division of Radiation Oncology, Department of Oncology, Cross Cancer Institute, University of Alberta, Edmonton, Canada; 20 Department of Urology, National Tokyo Medical Center, Tokyo, Japan; 21 National Institute of Radiological Science, National Institutes for Quantum and Radiological Science and Technology, Chiba, Japan; 23 Division of Genetics and Epidemiology, Institute of Cancer Research and Royal Marsden NHS Foundation Trust, London, UK; 24 Departments of Basic Medical Sciences and Radiotherapy, Ghent University Hospital, Ghent, Belgium; 25 Grupo de Medicina Xenómica, Centro de Investigación Biomédica en Red de Enfermedades Raras (CIBERER), Universidade de Santiago de Compostela, Santiago de Compostela, Spain; 26 Departments of Radiation Oncology & Genetics and Genomic Sciences, Icahn School of Medicine at Mount Sinai, New York, NY; 27 Department of Pathology and Cell Biology, Columbia University, New York, NY

## Abstract

**Background:**

A total of 10%–20% of patients develop long-term toxicity following radiotherapy for prostate cancer. Identification of common genetic variants associated with susceptibility to radiotoxicity might improve risk prediction and inform functional mechanistic studies.

**Methods:**

We conducted an individual patient data meta-analysis of six genome-wide association studies (n = 3871) in men of European ancestry who underwent radiotherapy for prostate cancer. Radiotoxicities (increased urinary frequency, decreased urinary stream, hematuria, rectal bleeding) were graded prospectively. We used grouped relative risk models to test associations with approximately 6 million genotyped or imputed variants (time to first grade 2 or higher toxicity event). Variants with two-sided *P*_meta_ less than 5 × 10^−8^ were considered statistically significant. Bayesian false discovery probability provided an additional measure of confidence. Statistically significant variants were evaluated in three Japanese cohorts (n = 962). All statistical tests were two-sided.

**Results:**

Meta-analysis of the European ancestry cohorts identified three genomic signals: single nucleotide polymorphism rs17055178 with rectal bleeding (*P*_meta_ = 6.2 × 10^−10^), rs10969913 with decreased urinary stream (*P*_meta_ = 2.9 × 10^−10^), and rs11122573 with hematuria (*P*_meta_ = 1.8 × 10^−8^). Fine-scale mapping of these three regions was used to identify another independent signal (rs147121532) associated with hematuria (*P*_conditional_ = 4.7 × 10^−6^). Credible causal variants at these four signals lie in gene-regulatory regions, some modulating expression of nearby genes. Previously identified variants showed consistent associations (rs17599026 with increased urinary frequency, rs7720298 with decreased urinary stream, rs1801516 with overall toxicity) in new cohorts. rs10969913 and rs17599026 had similar effects in the photon-treated Japanese cohorts.

**Conclusions:**

This study increases the understanding of the architecture of common genetic variants affecting radiotoxicity, points to novel radio-pathogenic mechanisms, and develops risk models for testing in clinical studies. Further multinational radiogenomics studies in larger cohorts are worthwhile.

Long-term side effects following radiotherapy affect the health-related quality of life for cancer survivors (1). Radiation dose and irradiated volume are the most important factors affecting toxicity risk but are broadly similar within patient populations. Known modifiers of the relationship between radiation exposure and risk of radiotoxicity include patient age, smoking history, concurrent treatments, and comorbidities. However, considerable interindividual variation in radiotoxicity remains unexplained after allowing for dosimetric and patient-level factors. An individual’s response to radiation is a heritable trait as evidenced by the similar cellular radiosensitivity of related individuals (2), intraindividual correlation in tissue response to therapeutic radiation (3), and the observation that rare mutations in some genes increase risk of radiotoxicity (4). Evidence suggests common variants may explain some of the remaining interindividual variation in radiotoxicity susceptibility (2,5). Simulation shows that the accuracy of models to predict radiotoxicity is improved when genetic risk variants are combined with clinical and dosimetric parameters (6).

Genetic predisposition to radiotoxicity in nonsyndromic individuals is poorly understood. Preclinical studies suggest that the biologic mechanisms are complex, involving cell death, premature senescence, inflammation, tissue remodeling with development of fibrosis, and vascular damage. Large genetic studies are difficult because it is challenging to build cohorts associated with a phenotype that takes months to years to develop. In addition, the radiosensitivity phenotype varies by tumor site and means of assessment.

In prostate cancer patients, common radiotoxicities include increased urinary frequency, radiation cystitis (characterized by urinary bleeding, pain, and inflammation), urinary retention, and rectal bleeding (7,8). Radiotoxicity prevalence in prostate cancer survivors varies by radiation delivery modality, time of follow-up, and method of symptom assessment (particularly for clinician vs patient scores). A large (n = 1571) prospective cohort study assessing clinician-assigned toxicity using the National Cancer Institute Common Terminology Criteria for Adverse Events reported the actuarial likelihood at 10 years of 15% and 9% for grade 2 or higher urinary and rectal toxicities, respectively (9).

Despite approximately 50% of cancer patients undergoing radiotherapy, the collection of radiotoxicity data is sporadic and inconsistent. Most centers do not collect data routinely with the detail and standardization required to conduct radiogenomic studies. There is also a clear potential for clinical impact, for example, with an option for modified radiotherapy planning as increasingly conformal dose delivery techniques become available. The Radiogenomics Consortium was established to facilitate data sharing and develop methods for meta-analyses. Our first pooled genome-wide association study (GWAS) performed in prostate cancer survivors demonstrated that it was possible to meta-analyze data and identify genomic risk regions despite the heterogeneity inherent in radiotherapy cohorts (11).

As part of a long-term goal to identify sufficient variants to develop a polygenic risk model for radiotoxicity, we undertook a larger meta-analysis on six cohorts comprising 3871 prostate cancer survivors. Secondary aims were to validate previously published single nucleotide polymorphisms (SNPs) and evaluate risk SNPs in Japanese cohorts. STROGAR guidelines (12) for reporting radiogenomic studies, which build on the STREGA and STROBE guidelines (13,14), were followed throughout.

## Methods

### Study Participants

The study included individuals with prostate adenocarcinoma, treated with radiotherapy with curative intent, and followed prospectively for development of toxicity. All participants gave written informed consent, and cohorts were collected following standards indicated by the Declaration of Helsinki. Participants were from either hospital-based cohorts or clinical trials (Supplementary Methods, available online). Supplementary Figure 1 (available online) summarizes the six mainly European-ancestry cohorts (Radiogenomics: Assessment of Polymorphisms for Predicting the Effects of Radiotherapy [RAPPER], RADIOGEN, Genetic Predictors of Adverse Radiotherapy Effects [GenePARE], University of Ghent [UGhent], Cross Cancer Institute–Brachytherapy [CCI-BT], Cross Cancer Institute–External Beam Radiotherapy [CCI-EBRT]) included in the meta-analysis and three Japanese cohorts (PRRG-photon, n = 170; PRRG-Cion, n = 538; and National Tokyo Medical Center (NTMC), n = 254; total n = 962) analyzed as a separate replication set. Individuals were excluded if DNA samples or genotyping failed quality control measures; they had non-European (or non-Japanese) ancestry determined using a preselected panel of ancestry informative markers (15); and/or data were unavailable on androgen-deprivation therapy, prior prostatectomy, age at treatment, and total biological effective dose (BED). A previously published GWAS meta-analysis (11) included a subset of RAPPER (RAPPER-I), RADIOGEN, GenePARE (GenePARE-I), and CCI-EBRT. RAPPER-II, GenePARE-II, UGhent, CCI-BT, and the Japanese cohorts were not analyzed previously.

### Assessment of Late Radiotherapy Toxicity

Toxicity was assessed from 6 months up to 5 years after the start of radiotherapy, with the exception of the UGhent cohort (follow-up from 18 to 30 months only). Different toxicity assessments were used (Supplementary Table 1, available online). Toxicity grades were harmonized to the Common Terminology Criteria for Adverse Events scale (Supplementary Table 1, available online). Assessment times were coarsened into discrete intervals for time-to-event analysis. Six-month intervals were used except for RAPPER, which used 12-month intervals after the first 2 years. Four toxicity outcomes were analyzed: increased urinary frequency, decreased urinary stream, hematuria, and rectal bleeding. We also tested a measure of overall toxicity using STAT score (21).

### Genotyping, Quality Control, and Imputation

Germline DNA from whole blood was genotyped for published GWASs (5,10,22–25) using Affymetrix SNP6.0 arrays (Affymetrix, Inc; CCI-EBRT and GenePARE-I) or Illumina CytoSNP12 arrays (Illumina, Inc; RAPPER-1). Everything else was genotyped using Illumina OncoArray-500K BeadChips (Illumina, Inc). Methods used by PRACTICAL were followed (15). Datasets were processed and imputed using reference haplotypes from the 1000 Genomes Project (see Supplementary Methods, available online).

### Statistical Analysis: GWAS Meta-Analysis

Genetic variants were tested for associations with toxicity using a grouped relative risk model adjusting for androgen-deprivation therapy, prior prostatectomy, age at treatment, and total BED. Covariables were selected a priori to reduce heterogeneity. A per-allele additive genetic model was assumed. In RAPPER (26) and GenePARE (23,27), where samples were genotyped in two batches using different arrays, batch was included as a binary adjustment variable. Outcomes were defined as time from the start of radiation to onset of first occurrence of grade 2 or higher toxicity (see Supplementary Table 1, available online) with time coarsened into discrete intervals. Efron method was used to handle ties.

A fixed-effects meta-analysis using inverse variance weighting combined genetic variant-toxicity associations across studies. Variants were considered statistically significant if the two-sided meta-analysis *P* value (*P*_meta_) was less than 5 × 10^-^^8^ and the chi-squared test of heterogeneity *P* value was greater than .05. The likelihood that an association is nominally statistically significant at a given threshold depends not only on the *P* value but also on the power to detect a given association. We therefore assessed the likelihood that our statistically significant associations represent false positives using the Bayes false discovery probability (28). Supplementary Table 2 (available online) reports power analysis for a range of minor allele frequencies and per-allele effect sizes. All statistical tests were two-sided.

### Fine-Scale Mapping and Credible Causal Variants (CCV) Annotation

Genomic regions surrounding each statistically significant association were fine-mapped using conditional analysis (details in Supplementary Materials, available online). CCVs were annotated with Variant Effect Predictor (31) to determine their effect on genes, transcripts, and protein sequences and overlapped with Encode enhancer-like and promoter-like regions (31–33). Potential expression Quantitative Trait Loci were identified using the Genotype Tissue Expression Portal.

### Statistical Analysis: Multivariable Modeling

Clinical and genetic variables were combined using cohort-stratified grouped relative risk models. Stepwise model selection was used to identify parsimonious multivariable models for each toxicity outcome. Confidence intervals and *P* values were likelihood based and two-sided, with *P* values of .05 or less considered statistically significant. The proportional hazards assumption for each predictor was tested at the nominal two-sided .05 level, one at a time, by extending the model to allow separate hazard ratios before and after 1095 days via an interaction of each predictor with I(time > 1095 days); all *P* values were greater than .05. Additional details are in the Supplementary Methods (available online).

## Results

**Figure 1.**
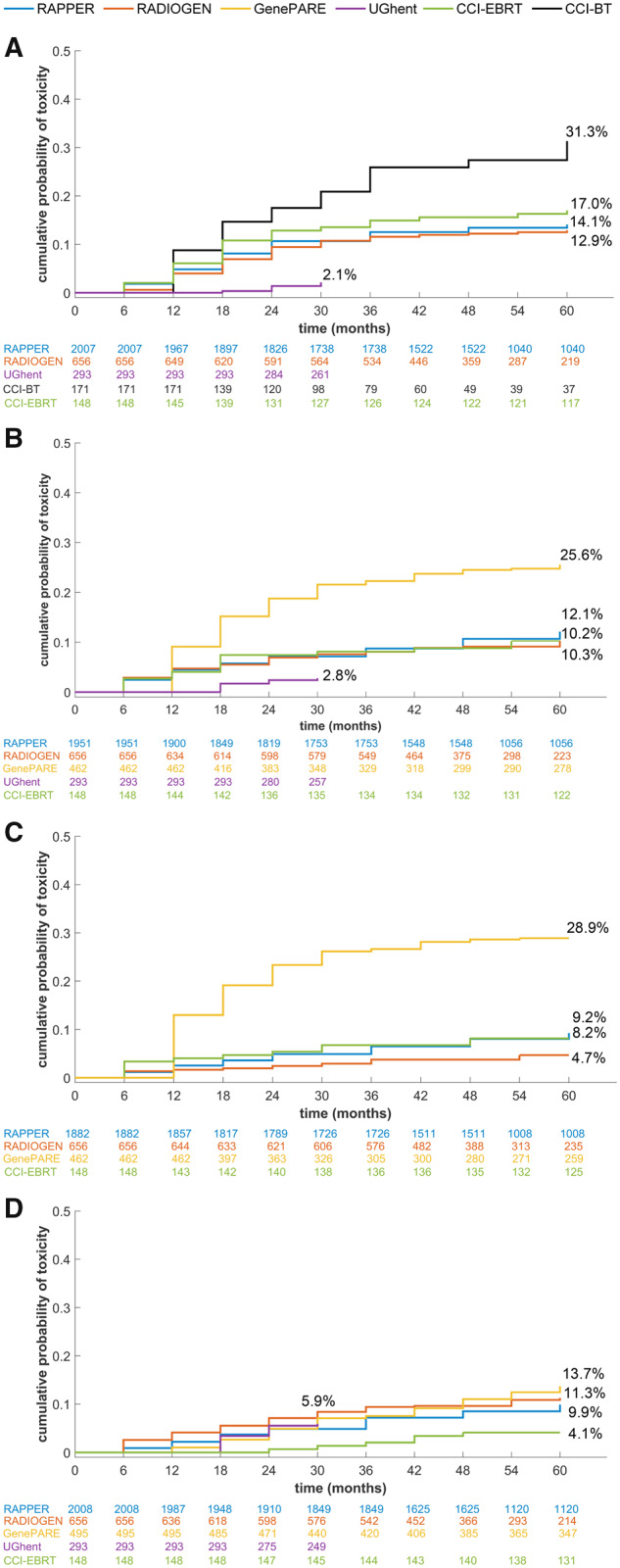
Cumulative probability of radiotoxicity. Each graph shows the cumulative probability of developing grade 2 or worse radiotoxicity for each individual outcome within each study included in the genome-wide association study meta-analysis. These outcomes include (**A**) rectal bleeding, (**B**) increased urinary frequency, (**C**) decreased urinary stream, and (**D**) hematuria. **Numbers** listed below the *x*-axis for each graph represent the numbers of patients at risk.

**Figure 2.**
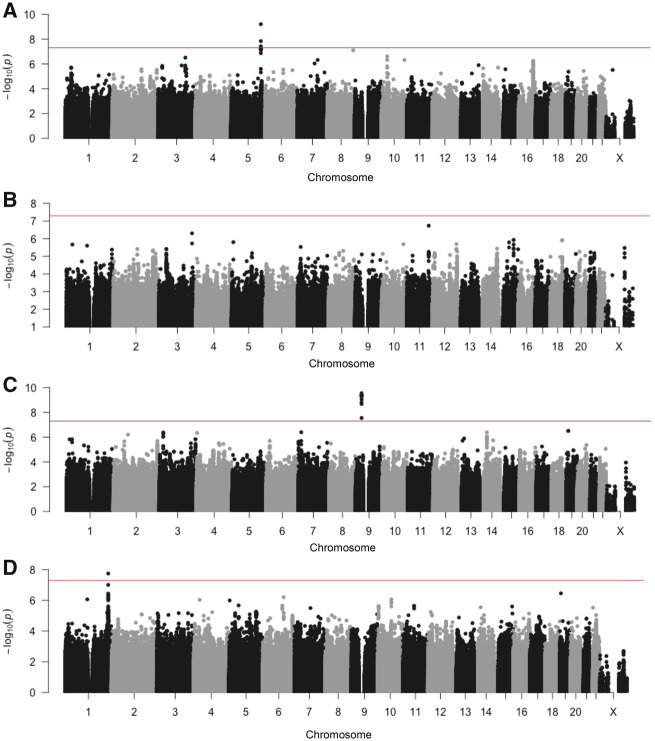
Manhattan plots. The graphs show association results for (**A**) rectal bleeding, (**B**) increased urinary frequency, (**C**) decreased urinary stream, and (**D**) hematuria. The **red line** denotes -log *P* value = 5 × 10^−8^. Each **point** represents a single nucleotide polymorphism, with **numbers** on the *x*-axis denoting chromosome number.

Table 1 summarizes the characteristics of the 3871 participants included in the meta-analysis. The cumulative probability of developing radiotoxicity varied by cohort and outcome (Figure 1; Table 1) and agreed with prior reports (9,37–40). As expected, radiotoxicity was higher in patients receiving brachytherapy (1). Lower toxicities were observed in UGhent, possibly due to differences in grading and/or shorter follow-up time. Bivariate Cox models provide evidence that the three urinary toxicities were associated with each other (eg, hazard ratio [HR] = 6.01, 95% confidence interval [CI] = 4.70 to 7.62 for association of decreased urinary stream with increased urinary frequency), whereas no urinary toxicity was associated with rectal bleeding (eg, HR = 0.96, 95% CI = 0.52 to 1.60 for association of decreased urinary stream with rectal bleeding). Meta-analysis Q-Q plots (Supplementary Figure 2, available online) showed no genomic inflation, suggesting population structure was adequately controlled using principal components analysis to restrict the study to European-ancestry individuals. Three independent SNPs reached statistical significance with a BFDP less than 2% (Table 2; Figure 2): rs17055178 with rectal bleeding (*P*_meta_ = 6.2 × 10^−^^10^), rs10969913 with decreased urinary stream (*P*_meta_ = 2.9 × 10^−^^10^), and rs11122573 with hematuria (*P*_meta_ = 1.8 × 10^−^^8^). Variants with minor allele frequency 1%–4% and SNPs falling just short of our criteria, including several variants associated with a secondary endpoint of overall toxicity measured using STAT score (21), are listed in Supplementary Table 3 (available online).

**Table 1.** Patient characteristics by cohort for the 3871 individuals included in the GWAS meta-analysis

Characteristics	All cohorts	RAPPER	RADIOGEN	GenePARE	UGhent	CCI-BT	CCI-EBRT
	(n = 3871)	(n = 2010)	(n = 658)	(n = 492)	(n = 311)	(n = 252)	(n = 148)
Age at treatment, median (range), y*	68 (43–86)	68 (48–84)	72 (47–86)	65 (43–85)	65 (49–81)	65 (45–79)	68 (45–82)
NCCN risk group, No. (%)							
Very low	545 (14.1)	133 (6.6)	100 (15.2)	172 (35.0)	43 (13.8)†	89 (35.3)	8 (5.4)
Low	258 (6.7)	82 (4.1)	23 (3.5)	61 (12.4)	21 (6.8)	68 (27.0)	3 (2.0)
Intermediate	2635 (68.1)	1566 (77.9)	447 (67.9)	232 (47.2)	173 (55.6)	95 (37.7)	122 (82.4)
High or very high	410 (10.6)	229 (11.4)	82 (12.5)	27 (5.5)	57 (18.4)	0	15 (10.1)
Not available	23 (0.6)	0	6 (0.9)	0	17 (5.5)	0	0
Stage at diagnosis, No. (%)							
T1a–c, T1x	1443 (37.3)	709 (35.3)	226 (34.3)	249 (50.6)	101 (32.5)	119 (47.2)	38 (25.7)
T2a–c, T2x	2020 (52.2)	1084 (53.9)	362 (55.0)	227 (46.1)	126 (40.5)	132 (52.4)	89 (60.1)
T3a–c, T3x	305 (7.9)	182 (9.1)	54 (2.7)	16 (3.3)	37 (11.9)	0	16 (10.8)
T4	14 (0.4)	0	7 (1.1)	0	6 (1.9)	0	1 (0.7)
Not available	89 (2.3)	35 (1.7)	9 (1.4)	0	41 (13.2)	1 (0.4)	4 (2.7)
Gleason at diagnosis, No. (%)							
≤6	1702 (44.0)	605 (30.1)	403 (61.2)	310 (63.0)	142 (45.7)	212 (84.1)	30 (20.3)
7	1653 (42.7)	1109 (55.2)	176 (26.8)	124 (25.2)	107 (34.4)	40 (15.9)	97 (65.5)
≥8	265 (6.8)	56 (2.8)	70 (10.6)	58 (11.8)	60 (19.3)	0	21 (14.2)
Not available	251 (6.5)	240 (11.9)	9 (1.4)	0	2 (0.6)	0	0
Pretreatment PSA, median (range)	8.9 (0–236.0)	10.1 (0.6–33.5)	9.7 (0.6–236.0)	6.2 (0.6–124.0)	6.6 (0–150.0)‡	6.3 (0.5–16.0)	10.9 (1.4–80.0)
Radical prostatectomy, No. (%)*							
Yes	225 (5.8)	0	128 (29.5)	0	97 (31.2)	0	0
No	3646 (94.2)	2010 (100)	530 (80.5)	492 (100)	214 (68.8)	252 (100)	148 (100)
Androgen-deprivation therapy, No. (%)*							
Yes	3047 (78.7)	2010 (100)	463 (70.4)	248 (50.4)	198 (63.7)	55 (21.8)	73 (49.3)
No	824 (21.3)	0	195 (29.6)	244 (49.6)	113 (36.3)	197 (78.2)	75 (50.7)
Type of radiotherapy, No. (%)							
3D-CRT	895 (25.4)	237 (11.8)	658 (100)	0	0	0	0
IMRT	2239 (57.8)	1773 (88.2)	0	7 (1.4)	311 (100)	0	148 (100)
Brachytherapy	534 (13.8)	0	0	282 (57.3)	0	252 (100)	0
Brachytherapy + EBRT	203 (5.2)	0	0	203 (41.3)	0	0	0
Total BED§, median (range)*	123 (52–292)	120 (107–123)	123 (57–127)	192 (52–269)	136 (124–136)	158 (80–292)	121 (112–134)
No. (%) with grade 2 or worse toxicity							
Increased urinary frequency‖	436 (11.5)	219 (10.9)	60 (9.1)	113 (24.6)	8 (2.6)¶	NA#	15 (10.1)
Decreased urinary stream**	345 (9.9)	159 (7.9)	27 (4.1)	125 (27.2)	NA††	NA#	12 (8.1)
Hematuria‡‡	333 (9.2)	182 (9.1)	66 (10.0)	62 (12.6)	17 (5.5)¶	NA§§	6 (4.1)
Rectal bleeding‖‖	423 (12.5)	273 (13.6)	79 (12.0)	NA¶¶	6 (1.9)¶	40 (15.9)	25 (16.9)

* Age at treatment, radical prostatectomy, androgen-deprivation therapy, and total BED were included as covariates in the GWAS meta-analysis. BED = biological effective dose; CCI-BT = Cross Cancer Institute–Brachytherapy; CC-EBRT = Cross Cancer Institute–External Beam Radiotherapy; 3D-CRT = three-dimensional conformal radiotherapy; EBRT = external beam radiotherapy (either 3D-CRT or IMRT); GenePARE = Genetic Predictors of Adverse Radiotherapy Effects; GWAS = genome-wide association study; IMRT = intensity-modulated radiotherapy; NCCN = National Comprehensive Cancer Network; PSA = prostate specific antigen; RAPPER = Radiogenomics: Assessment of Polymorphisms for Predicting the Effects of Radiotherapy; UGhent = University of Ghent.

† NCCN risk group in the UGhent cohort was defined using preradiotherapy PSA rather than PSA at diagnosis.

‡ PSA measurement is pre-radiotherapy but postprostatectomy in patients who received prior prostatectomy.

§ Total BED was calculated using an α to β ratio of 3 following Ho et al. (27).

‖ Increased urinary frequency was evaluable in 3782 participants with available baseline and follow-up data (2010 in RAPPER, 658 in RADIOGEN, 459 in GenePARE, 303 in UGhent, and 148 in CCI-EBRT).

¶ Follow-up in UGhent was from 18 months to 30 months as opposed to 6 months to 5 years in all other studies.

# Increased urinary frequency and decreased urinary stream were not analyzed in CCI-BT because assessments were not conducted at regular intervals.

** Decreased urinary stream was evaluable in 3470 participants with available baseline and follow-up data (2010 in RAPPER, 658 in RADIOGEN, 459 in GenePARE, and 148 in CCI-EBRT).

†† Decreased urinary stream was not assessed in UGhent.

‡‡ Hematuria was evaluable in 3619 participants with available baseline and follow-up data (2010 in RAPPER, 658 in RADIOGEN, 492 in GenePARE, 311 in UGhent, and 148 in CCI-EBRT).

§§ Hematuria was not assessed in CCI-BT.

‖‖ Rectal bleeding was evaluable in 3379 participants with available baseline and follow-up data (2010 in RAPPER, 658 in RADIOGEN, 311 in UGhent, 252 in CCI-BT, and 148 in CCI-EBRT).

¶¶ Rectal bleeding was assigned a single grade in GenePARE using information across all follow-up assessments, so this outcome was not available for analysis.

**Table 2.** Study-specific and overall results for new risk SNPs identified via GWAS meta-analysis* of six European ancestry cohorts

Genetic variant	Chr†	Minor allele	MAF‡	Toxicity outcome	Study	Info§	Mean minor allele dosage		HR (95% CI)‖	*P*_meta_ ¶	*P*_het_ #	BFDP**, %
							Toxicity	No toxicity				
rs17055178	chr5: 157, 403, 410	G	0.09	Time to first grade 2+ rectal bleeding	Meta-analysis	–			1.95 (1.58 to 2.40)	6.2 × 10^−10^	.61	0.09
					RAPPER	0.81, 0.99	0.22	0.13	1.78 (1.37 to 2.32)			
					RADIOGEN	0.99	0.33	0.14	2.58 (1.69 to 3.95)			
					GenePARE	NA††	NA††	NA††	NA††			
					UGhent‡‡	0.99	0.17	0.14	1.38 (0.18 to 10.4)			
					CCI-BT	0.99	0.25	0.14	2.01 (0.97 to 4.20)			
					CCI-EBRT	0.98	0.12	0.13	1.27 (0.38 to 4.25)			
rs10969913	chr9: 30, 866, 808	G	0.05	Time to first grade 2+ decreased urinary stream	Meta-analysis	–			3.92 (2.57 to 6.00)	2.9 × 10^−10^	.08	1.07
					RAPPER	0.61, 0.95	0.04	0.02	1.86 (0.76 to 4.54)			
					RADIOGEN	0.95	0.04	0.02	2.03 (0.27 to 15.4)			
					GenePARE	0.99, 0.95	0.11	0.04	4.36 (2.55 to 7.46)			
					UGhent	NA§§	NA§§	NA§§	NA§§			
					CCI-BT	NA‖‖	NA‖‖	NA‖‖	NA‖‖			
					CCI-EBRT	0.95	0.27	0.02	14.3 (3.78 to 54.4)			
rs11122573	chr1: 230, 837, 180	T	0.06	Time to first grade 2+ hematuria	Meta-analysis	–			1.92 (1.53 to 2.42)	1.8 × 10^−8^	.14	1.96
					RAPPER	0.99	0.19	0.14	1.42 (0.99 to 2.04)			
					RADIOGEN	0.99	0.34	0.15	2.40 (1.54 to 3.73)			
					GenePARE	0.99, 0.99	0.18	0.11	2.01 (1.25 to 3.22)			
					Ughent	0.99	0.47	0.14	3.59 (1.72 to 7.49)			
					CCI-BT	NA¶¶	NA¶¶	NA¶¶	NA¶¶			
					CCI-EBRT	1.000	0.17	0.16	0.99 (0.13 to 7.58)			

* Within each cohort, SNP-toxicity associations were adjusted for age at treatment, prior prostatectomy, adjuvant hormonal therapy, and total BED. Associations in RAPPER and GenePARE were also adjusted for genotyping batch. Bold values correspond to results from meta-analysis. BED = biological effective dose; BFDP = Bayesian false discovery probability; CCI-BT = Cross Cancer Institute–Brachytherapy; CC-EBRT = Cross Cancer Institute–External Beam Radiotherapy; Chr = chromosome; CI = confidence interval; GenePARE = Genetic Predictors of Adverse Radiotherapy Effects; GWAS = genome-wide association study; HR = hazard ratio; MAF = minor allele frequency; NA = not analyzed; *P*_meta_ = meta-analysis *P* value; *P* = heterogeneity *p* value; RAPPER = Radiogenomics: Assessment of Polymorphisms for Predicting the Effects of Radiotherapy; SNP = single nucleotide polymorphism; UGhent = University of Ghent.

† Base position is according to Genome Reference Consortium Human Build 37 (hg19).

‡ Minor allele frequency for each is from PRACTICAL Oncoarray samples of European ancestry.

§ Imputation info score values in RAPPER are from the cytoSNP12 Array and Oncoarray, respectively; values in Gene-PARE are from the AffySNP6.0 array and OncoArray, respectively; values in all other studies are from the OncoArray.

‖ Hazard ratio corresponds to the minor allele with the major allele treated as the reference group.

¶ Two-sided *P*_meta_ was calculated using a Wald test.

# Two-sided heterogeneity *P* value was calculated using a χ^2^ test.

** BFPD estimated assuming a prior variance, W = 0.32^2^, and prior probability of a non-null association .0001.

†† Rectal bleeding was assigned a single grade in GenePARE using information across all follow-up assessments, so this outcome was not available for analysis using time-to-event analysis.

‡‡ There were only six rectal bleeding events in UGhent. Exclusion of this cohort from meta-analysis had minimal impact on the results: HR = 1.95, 95% CI = 1.58 to 2.41, *P*_meta_ 6.1 × 10^−10^.

§§ Decreased urinary stream was not assessed in UGhent.

‖‖ Increased urinary frequency and decreased urinary stream were not analyzed in CCI-BT because assessments were not conducted at regular intervals.

¶¶ Hematuria was not assessed in CCI-BT.

**Figure 3.**
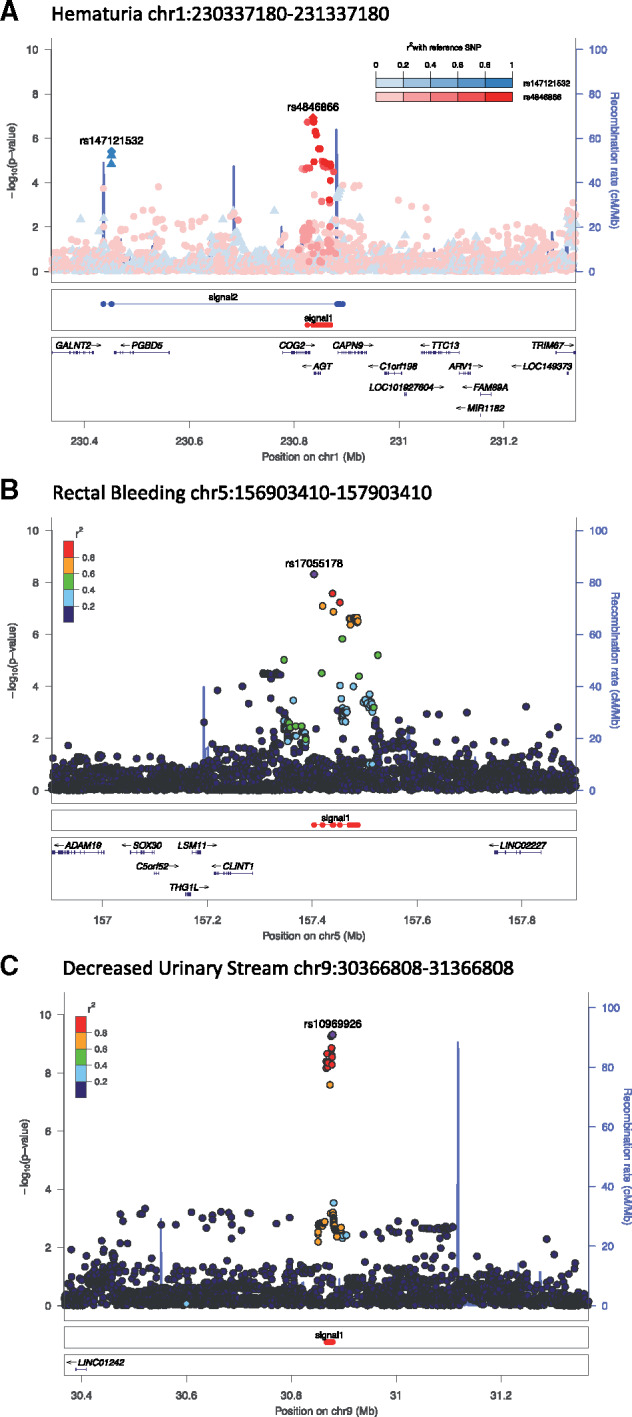
Regional Manhattan plots. The graphs show signals defined by fine-mapping of the (**A**) hematuria risk region chromosome (chr): 230337180–231337180, (**B**) rectal bleeding risk region chr5: 156903410–157903410, and (**C**) decreased urinary stream risk region chr9: 30366808–31366808.

Fine-scale mapping identified CCVs (Figure 3; Supplementary Tables 4 and 5, available online). A second independent signal (tagged by rs147121532, *P*_conditional_ = 4.7 × 10^−^^6^) at chromosome (chr)1: 230337180–231337180 was associated with hematuria. The first signal (tagged by rs11122573, Supplementary Tables 4 and 5, available online) includes 47 CCVs (together explaining 93% of the posterior probability of risk). These CCVs lie in active enhancer- or promoter-like gene-regulatory regions (Supplementary Figure 3A; Supplementary Table 6, available online). Their risk alleles decrease expression of *AGT* (encoding angiotensinogen; ENSG00000135744.7) and *COG2* (encoding conserved oligomeric Golgi complex subunit 2; ENSG00000135775.9) in multiple tissues including arteries (Supplementary Figure 3B, available online). The second signal with 10 CCVs (tagged by rs147121532, explaining 54% of the posterior probability; Figure 3; Supplementary Tables 4 and 5, available online) has risk alleles that decrease expression of *CAPN9* (encoding the intestinal protease, calpain-9; ENSG00000135773.8) and *ARV1* (encoding ARE2 required for viability [ARV1] homolog, fatty acid homeostasis modulator; ENSG00000173409.9). The risk alleles in the second signal were also associated with differential expression of two noncoding (nc) RNAs: decreased expression of ncRNA AL512328.1 (ENSG00000244137.1), which overlaps partially with *AGT* and *CAPN9*, and increased expression of ncRNA LOC101927604 (ENSG00000223393.1). At the chr5: 156903410–157903410 region associated with rectal bleeding, a 15 CCV signal accounts for 98% posterior probability at the region (Figure 3; Supplementary Tables 4 and 5, available online). CCVs in this region overlap active enhancer-like regions in gastrointestinal tissues (Supplementary Figure 3A; Supplementary Table 6, available online), but none were statistically significantly associated with differential gene expression in GTEx (all *P* > .05). At the chr9: 30366808–31366808 region, associated with decreased urinary stream, a single 15 CCV signal accounts for 99% of the posterior probability (Figure 3; Supplementary Tables 4 and 5, available online). None were statistically significantly associated with differential gene expression in the tissues evaluated by GTEx (all *P* > .05).

To explore biological mechanisms underpinning radiotoxicity, we computed gene and pathway scores from the meta-analysis results (Supplementary Tables 7 and 8, available online). Nine pathways were associated (*P* < .05) with multiple toxicities (Supplementary Table 8, available online), suggesting a common biological mechanism. For example, the extracellular matrix pathway from the Biocarta database was associated with increased urinary frequency as well as hematuria; “cytokine signaling in immune system” from the Reactome database was associated both with decreased urinary stream and hematuria.

SNPs previously associated with radiotoxicity were evaluated for replication in the new cohorts (Table 3). Three SNPs showed a consistent association signal (rs17599026 with increased urinary frequency, rs7720298 with decreased urinary stream, and rs1801516 with overall toxicity), although the effect size was attenuated. SNPs in *TANC1* (22) showed inconsistent results.

**Table 3.** Association results for risk loci identified in prior genetic association studies

Genetic variant and gene symbol	Chr†	Minor allele	MAF‡	Toxicity outcome	Results from prior publication			Meta-analysis of new studies not included in prior publication			
					OR (95% CI)	*P*_meta_ §	No.	Study, No.	Info*	OR (95% CI)	*P*_meta_ §
rs17599026	chr5: 137,763, 798	T	0.07	Presence of grade 1+ increased urinary frequency at 2 years after radiotherapy	3.12	4.2 × 10^−8^	1564	Meta-analysis	–	1.23 (0.91 to 1.67)	.19
KDM3B					(2.08 to 4.69)			RAPPER-II, n = 1255	0.96	1.27 (0.90 to 1.80)	
								GenePARE-II, n = 161	0.96	1.10 (0.45 to 2.69)	
								UGhent, n = 281	0.96	1.08 (0.44 to 2.64)	
rs7720298	chr5: 13, 858, 328	G	0.30	Presence of grade 1+ decreased urine stream at 2 years after radiotherapy	2.71	3.2 × 10^−8^	1564	Meta-analysis	–	1.37 (1.01 to 1.86)	.05
DNAH5					(1.90 to 3.86)			RAPPER-II, n = 1255	0.98	1.27 (0.88 to 1.83)	
								GenePARE-II, n = 161	0.98	1.61 (0.92 to 2.82)	
rs1801516	chr11: 108, 175, 462	A	0.22	Overall toxicity‖¶	1.21	NR	2697	Meta-analysis	–	1.37 (1.05 to 1.78)	.02
ATM					(0.98 to 1.49)			RAPPER-II, n = 859	NA#	1.36 (1.03 to 1.80)	
								GenePARE-II, n = 101	NA#	1.45 (0.63 to 3.34)	
								CCI-BT, n = 82	NA#	1.18 (0.21 to 6.55)	
rs7582141	chr2: 159, 899, 489	T	0.02 to 0.05††	Overall toxicity¶	6.17	4.2 × 10^−10^	1742	Meta-analysis	–	0.98 (0.52 to 1.86)	.95
TANC1					(2.25 to 16.9)			RAPPER-II, n = 1340	0.96	0.56 (0.20 to 1.59)	
								GenePARE-II, n = 220	0.96	0.85 (0.20 to 3.67)	
								UGhent, n = 285	0.96	2.16 (0.71 to 6.53)	
								CCI-BT, n = 114	0.96	NA**	
								CCI-EBRT, n = 148	NA#	0.73 (0.08 to 6.38)	

* Imputation info score values in CCI-EBRT are from the AffySNP6.0 array; values in all other studies are from the OncoArray. CCI-BT = Cross Cancer Institute–Brachytherapy; CC-EBRT = Cross Cancer Institute–External Beam Radiotherapy; Chr = chromosome; CI = confidence interval; GenePARE = Genetic Predictors of Adverse Radiotherapy Effects; MAF = minor allele frequency; NA = not analyzed; NR = not reported; OR = odds ratio; RAPPER – Radiogenomics: Assessment of Polymorphisms for Predicting the Effects of Radiotherapy; SNP = single nucleotide polymorphism; UGhent = University of Ghent.

† Base position according to Genome Reference Consortium Human Build 37 (hg19).

‡ Minor allele frequency is from PRACTICAL OncoArray samples of European ancestry.

§ Two-sided *P*_meta_ was calculated using a Wald test.

‖ The previously published study included acute as well as late toxicity whereas the current study includes only late toxicity.

¶ Overall toxicity was measured using STAT score (21) based on the worst toxicity grade from 2 years to 5 years after the start of radiotherapy. Analysis is adjusted for preradiotherapy STAT score, age, androgen-deprivation therapy, prostatectomy, and total biological effective dose. Analysis in RADIOGEN used genotype data from the Illumina OncoArray whereas the previously published results used genotype data from the Affymetrix Axiom Genome-Wide CEU 1 array (22). Additional toxicity follow-up data were available in the current analysis that were not available in the earlier analysis.

# SNP was directly genotyped.

** STAT score was not assessed in CCI-BT because it correlated perfectly with rs7582141 genotype.

†† The minor allele frequency for rs7582141 and other SNPs in this locus vary across European subpopulations. The frequency of the C allele is 0.024 in RAPPER-I, 0.022 in RAPPER-II, 0.039 in RADIOGEN, 0.042 in GenePARE-I, 0.046 in GenePARE-II, 0.029 in UGhent, and 0.033 in CCI-EBRT.

The new and previously published variants were evaluated in Japanese ancestry cohorts (Supplementary Table 9, available online). In NTMC, the minor alleles of four SNPs (rs10969913, rs17599026, rs7720298, rs7582141) were associated with a nonstatistically significantly increased risk of toxicity, with the effect direction consistent with observations in Europeans. The PRRG photon cohort also showed consistent, though nonstatistically significant, association signals for rs10969913 and rs17599026. Associations were not replicated in the PRRG carbon-ion cohort.

Multivariable grouped relative risk models identified independent clinical, dosimetric, and/or comorbidity factors associated with an increased hazard ratio for each toxicity outcome (Table 4). Importantly, SNPs remained independently associated with toxicity and with similar effect sizes in every model. The effect sizes for SNPs were similar to those of established dosimetric or patient-related risk factors. Adjustment for the first seven principal components derived from analysis of European ancestry samples had minimal impact on model parameters. C-statistics show that although model performance was modest, all four models improved slightly with the addition of SNPs (Supplementary Table 10, available online).

**Table 4.** Multivariable models including SNPs and clinical risk factors. All models are stratified by study

Model	HR (95% CI)	P*
Rectal bleeding†		
rs17055178	1.84 (1.49 to 2.24)	<.001
Rectum volume (cm^3^) receiving 65 Gy‡	1.33 (1.08 to 1.63)	.007
Rectum volume (percent) receiving 70 Gy§	1.44 (1.18 to 1.77)	<.001
Arthritis	2.06 (1.12 to 3.48)	.02
Inflammatory bowel diverticular disease	1.80 (1.07 to 2.83)	.03
Rectal dose standard deviation§	1.10 (1.03 to 1.18)	.008
Intestinal volume (percent) receiving 15 Gy¶	1.26 (1.03 to 1.52)	.03
Gleason score ≥7#	1.25 (1.00 to 1.57)	.05
Cardiovascular disease	1.44 (1.01 to 2.02)	.05
Increased urinary frequency**		
rs17599026	1.37 (1.08 to 1.71)	.01
Age at treatment >75††	1.50 (1.16 to 1.92)	.002
Diabetes	1.53 (1.15 to 2.00)	.005
Cardiovascular disease	1.57 (1.04 to 2.31)	.04
Prior pelvic surgery	1.57 (1.06 to 2.24)	.02
Presence of hemorrhoids	1.56 (1.02 to 2.27)	.04
Decreased urinary stream‡‡		
rs10969913	2.23 (1.36 to 3.44)	.002
rs7720298	1.25 (1.05 to 1.48)	.01
Presence of hemorrhoids	2.06 (1.29 to 3.13)	.004
Prior TURP	1.67 (1.13 to 2.39)	.01
Bladder volume (cm^3^) receiving 70 Gy§§	1.35 (1.09 to 1.87)	.002
Hematuria‖‖		
rs11122573	1.77 (1.39 to 2.23)	<.001
rs75991123¶¶	1.61 (1.22 to 2.09)	<.001
Prior TURP	2.33 (1.70 to 3.12)	<.001
Bladder volume (%) receiving 74 Gy##	1.29 (1.09 to 1.51)	.003
Receipt of EBRT***	1.92 (1.17 to 3.20)	.01
Age at treatment†††	2.80 (1.21 to 5.91)	.02

* Two-sided *P* value was calculated using a Wald test. EBRT = external beam radiotherapy; HR = hazard ratio; TURP = transurethral resection of the prostate.

† There were only six rectal bleeding events in UGhent so this cohort was excluded from the model.

‡ Variable was log2 transformed and includes a spline knot at 3.0 cm^3^, the 25th percentile value. Hazard ratio is per doubling of volume above the 25th percentile value, with reference being values below the 25th percentile.

§ Variable was log2 transformed and includes a spline knot at 1.7%, the 75th percentile value. Hazard ratio is per doubling of percent above the 75th percentile, with reference being values below the 75th percentile.

‖ This variable is defined as the SD from the mean rectal dose for the standardized rectal volume defined as a solid organ, for each individual patient’s dosimetry. It includes a spline knot at 19.7 Gy, the median value. Hazard ratio is per unit above the median value, with reference being values below the median.

¶ Variable was log2 transformed and includes a spline knot at 3.3%, the 75th percentile value. Hazard ratio is per doubling of percent above the 75th percentile, with reference being values below the 75th percentile.

# Reference group is Gleason less than 7.

** There were only eight increased urinary frequency events in UGhent so this cohort was excluded from the model.

†† Reference group are men 75 years or younger at time of treatment.

‡‡ There were only 12 decreased urinary stream events in CCI-EBRT so this cohort was excluded from the model.

§§ Variable was log2 transformed. Hazard ratio is per doubling of volume.

‖‖ There were only six hematuria events in CCI-EBRT so this cohort was excluded from the model.

¶¶ The top SNPs in the second region associated with hematuria, rs147121532, has a minor allele frequency less than 4% and so the next most strongly associated SNP was used in the multivariable model (minor allele frequency 6%).

## Variable was log2 transformed and includes a spline knot at 1.9 cm^3^, the median value. Hazard ratio is per doubling of volume above the median value, with reference being values below the median. In UGhent, bladder volume (percentage) receiving 75 Gy was used instead of bladder volume (percentage) receiving 74 Gy.

*** Reference group received brachytherapy alone.

††† Age is treated as a continuous variable if older than 75 years. Hazard ratio is per year of age older than 75 years, with reference being men age 75 years or younger.

## Discussion

Our study identified three new genomic regions associated with radiotoxicity. By performing the first radiogenomics fine-mapping study, we identified CCVs in each region and demonstrated two independent signals in the region associated with hematuria. The CCVs in this region were associated with differential expression of local protein coding genes (*AGT*, *COG2*, *CAPN9*, *ARV1*) and nc RNAs (AL512328.1, LOC101927604), pointing toward possible functional mechanisms. For example, *AGT* encoding angiotensinogen is converted to the active enzyme angiotensin II by angiotensin-converting enzyme (ACE). Prior studies suggest angiotensin signaling may influence radiation-induced blood vessel wall injury and interstitial fibrosis (41), and animal and human studies suggest that ACE inhibitors could be radioprotective (42–46). The regions associated with rectal bleeding and decreased urinary stream did not contain CCVs associated with differential expression of nearby genes in the available tissue databases and may associate with long-distance gene regulation. The rectal bleeding locus overlaps with active enhancer-like regions in gastrointestinal tissues, and further studies are needed to uncover the functional effects.

Pathway analysis might also identify new mechanisms involved in the pathogenesis of radiotoxicity. For example, the top-ranking pathway associated with hematuria was platelet adhesion to exposed collagen. Platelet adhesion is the first step in the formation of a platelet plug during hemostasis in response to blood vessel wall injury (47). Collagens are involved in the process and some are abundant in vascular epithelia (48). Several collagen-binding proteins are expressed on platelets including integrins (47). Interestingly, the integrin pathway ranked eighth for an association with rectal bleeding.

Our analysis is the largest GWAS of late radiotoxicity. Although heterogeneity across cohorts adds noise and masks statistically significant SNP-toxicity associations, our analysis plan addressed heterogeneity. Use of individual patient data meta-analysis maximizes statistical power when combining information across multiple studies (49,50). In addition, we stratified our multivariable models by study to account for differences in toxicity incidence and other unmeasured covariates that may differ across study populations. Analysis of previously identified risk SNPs provides support for the original discovery because the odds ratios, the best estimate of association, are in the same direction in the new studies. As is commonly observed, the initial effect size estimates for the prior GWAS-identified SNPs were upwardly biased (so-called “winner’s curse”), and evaluation here enabled estimation of effect sizes that more likely reflect their true contribution to risk of toxicity. Although unable to replicate the association at 2q24.1 within *TANC1*, it is challenging because of the rarity of minor alleles within Europeans (30). However, ongoing laboratory studies support a role of *TANC1* in radiation response (personal communication from Ana Vega, Universidade de Santiago de Compostela, Santiago de Compostela, Spain), highlighting the importance of functional studies as complementary to association studies.

The multivariable risk models demonstrate that genetic variants, treatment variables, and other clinical factors are independent predictors for radiotoxicity, supporting the suggestion that common variants can improve traditional normal tissue complication probability models (51,52). Although the c-statistics for our models are modest, SNPs improved model performance. As cohort and sample sizes increase, further GWAS should uncover additional risk SNPs and sequencing studies will uncover rare, possibly high-penetrance, variants. We foresee the eventual incorporation of polygenic scores or gene panel information into integrative models of radiotoxicity that also include clinical risk factors. Although important to continue efforts to identify additional risk SNPs and rare variants, our models are ready for validation, a critical next step toward clinical testing. The REQUITE prospective cohort study (53) of late radiotoxicity will provide an excellent opportunity to test these risk models. In addition, investigators are actively developing novel clinical trial approaches for testing the ability of risk models to personalize treatment and improve outcomes (54). We foresee these models being used, for example, to identify the small proportion of individuals with the highest risk for toxicity who might choose to avoid radiotherapy if watchful waiting or surgery are alternatives; for dose escalation in patients with a low risk of toxicity; and to evaluate how model-directed modification of planned doses to normal tissues affects risk for developing toxicity.

A strength of our study is the prospective longitudinal assessment of toxicity enabling use of time-to-event analysis to maximize information across multiple toxicity assessments. Long-term follow-up is clearly important for radiogenomic studies, and future work should use longitudinal analysis when possible. A second strength of our work is the inclusion of Japanese ancestry cohorts. Most GWAS to date focused on populations of European ancestry, because ethnicity inflates type I error rates and reduces statistical power because of population heterogeneity in allelic effects on a trait (55). It is important to understand how knowledge gained from European populations transfers to other ethnicities. Methods are being developed to detect genetic variants associated with complex traits allowing for population heterogeneity (56). Transethnic studies suggest susceptibility loci for traits are generally shared between European and East Asians (57), and, because of the larger sample size, cross-population meta-analyses increase statistical power to detect novel loci (58). Our cohort sizes are still too small to identify heterogeneity in allelic effects between ethnic groups. However, we performed the first analysis exploring transferability of SNP-radiotherapy toxicity associations across ethnicities.

Limitations include the lack of detailed dosimetry and comorbidity data for all cohorts, although our stratified analysis maximized use of data across all studies for multivariable models. In addition, although this is the largest GWAS of its kind to date, the sample size is modest compared with disease susceptibility GWAS and was powered only to detect SNP-toxicity associations with relatively large effect sizes. Our study was too small for multi-SNP modeling, such as polygenic risk score and machine learning-based methods. The latter were successful for other polygenic traits and diseases (59) and should be reevaluated as larger radiotoxicity cohorts become available. In addition, this study was not designed to identify rare variants associated with radiotherapy toxicity, which requires next-generation sequencing of large cohorts or case-control studies.

In summary, by performing the largest GWAS meta-analysis and first fine-mapping study in radiogenomics, to our knowledge, we identified four new regions associated with radiotoxicity in prostate cancer. We showed the signals affect gene regulation rather than gene coding sequences. This study increases our understanding of the architecture of common genetic variants affecting radiotoxicity and demonstrates that further multinational radiogenomics studies in larger cohorts are worthwhile.

## Funding

This work was supported by the US National Institutes of Health (NIH; K07 CA187546 to SLK; SBIR HHSN261201500043C to BSR), the United States Department of Defense (PC140371 to BSR and HO), and the European Union’s Horizon 2020 research and innovation programme under the Marie Sklodowska-Curie grant agreement No. 656144. Genotyping via the OncoArray was funded by the NIH (U19 CA 148537 for ELucidating Loci Involved in Prostate cancer SuscEptibility [ELLIPSE] project and X01HG007492 to the Center for Inherited Disease Research [CIDR] under contract number HHSN268201200008I) as well as C8197/A16565. Additional analytic support was provided by NIH NCI U01 CA188392 (PI: Schumacher). RAPPER was supported by Cancer Research UK grants C1094/A18504, C147/A25254, and C147/A25254 and NIHR Manchester Biomedical Research Centre funding. The PRACTICAL consortium was supported by Cancer Research UK Grants C5047/A7357, C1287/A10118, C1287/A16563, C5047/A3354, C5047/A10692, and C16913/A6135; European Commission’s Seventh Framework Programme grant agreement No. 223175 (HEALTH-F2-2009–223175); and the NIH Cancer Post-Cancer GWAS initiative grant: 1U19 CA 148537–01 (the GAME-ON initiative). CMLW is supported by the NIHR Manchester Biomedical Research Centre and the EU’s 7th Framework Programme Grant Agreement No 601826. GP and MEA’s work was also partially supported by NIH grant R01GM114434. RADIOGEN research was supported by Spanish Instituto de Salud Carlos III funding, an initiative of the Spanish Ministry of Economy and Innovation partially supported by European Regional Development FEDER Funds (INT15/00070, INT16/00154, INT17/00133; PI16/00046; PI13/02030; PI10/00164), and through the Autonomous Government of Galicia (Consolidation and structuring program: IN607B) given to AV. DPD was supported by the NIHR Royal Marsden Hospital and Institute of Cancer Research Biomedical Research Centre.

## Notes

The funders had no involvement in the study design, data collection, analysis and interpretation, writing of the report, or the decision to submit the paper for publication. The authors have no conflicts of interest directly related to this study to disclose.

The authors thank the Prostate Cancer Association Group to Investigate Cancer Associated Alterations in the Genome (PRACTICAL) Consortium (http://practical.icr.ac.uk/) for providing genotyping data for studies genotyped using the Oncoarray. We are grateful to the collaborating clinicians for participation in the PRRG study. This work was enabled by the computational resources and staff expertise provided by Scientific Computing at the Icahn School of Medicine at Mount Sinai and the University of Rochester Center for Integrated Research Computing (CIRC).

## Supplementary Material

djz075_Supplementary_Data
