# Erratum

**DOI:** 10.1093/jnci/djz237

**Published:** 2019-12-14

**Erratum to “Radiogenomics Consortium Genome-Wide Association Study Meta-Analysis of Late Toxicity After Prostate Cancer Radiotherapy” by Sarah L Kerns et al. *JNCI. J Natl Cancer Inst* (2019), doi: 10.1093/jnci/djz075.**

In this article, the name of Sarah L. Kerns should be displayed as Sarah L. Kerns*, indicating that Dr. Kerns contributed equally to this work. The Publisher regrets the error.

