# Supplementary material for: Radiogenomics Consortium Genome-Wide Association Study Meta-Analysis of Late Toxicity After Prostate Cancer Radiotherapy
Source: J Natl Cancer Inst. 2019 May 16;112(2):179–90. doi: 10.1093/jnci/djz075 (PMC7019089; doi:10.1093/jnci/djz075)
Supplement: djz075_Supplementary_Data [file djz075_supplementary_data.pdf]

Supplementary Methods

Study Participants

RAPPER (UKCRN1471; [1]) recruited participants enrolled in the RT01 (ISRCTN47772397 [2]) and CHHiP (ISRCTN97182923 [3, 4]) trials, and was approved by the Cambridge South Research Ethics Committee (05/Q0108/365). RADIOGEN recruited participants treated at the Clinical University Hospital of Santiago de Compostela, Spain and was approved by the Galician Ethical Committee [5]. Gene-PARE [6, 7] recruited participants treated at the Mount Sinai Hospital and was approved by the Mount Sinai Medical Center Institutional Review Board. UGhent recruited participants from the Ghent University Hospital [8] and was approved by the Ghent University Hospital ethics committee. CCI-BT and CCI-EBRT [9] were recruited from the Cross Cancer Institute and the Tom Baker Cancer Centre in Canada following approval by the Health Research Ethics Board of Alberta. PRRG participants were recruited at the Hospital of the National Institute of Radiological Sciences and received either external beam photon therapy (PRRG-photon) or carbon-ion therapy (PRRG-Cion). All the patients provided written informed consent to participate in the study between 2001 and 2010, which was approved by the Certified Review Board at the National Institute of Radiological Sciences (06-004) and by each collaborating institution (Tohoku University Hospital, Yokohama City University Hospital, Nagoya City University Hospital and Kyushu University Hospital). NTMC participants were recruited from the National Tokyo Medical Center and were treated with permanent seed brachytherapy with or without external beam photon therapy. All participants provided informed consent, and the study was approved by the local Institutional Review Board.

Assessment of late radiotherapy toxicity

Toxicity was assessed using the following: the Late Effects in Normal Tissue [10], Royal Marsden Hospital [11], and Radiation Therapy Oncology Group [12] scales (RAPPER); the NCI CTCAE [13] (RADIOGEN, CCI-EBRT, UGhent); and the American Urological Association Symptom Score [14] and an institutional scale (GenePARE). UGhent and CCI-BT used a simple measure of presence or absence for rectal bleeding.

Associations between pairs of toxicities were assessed by hazard ratios, considering each toxicity as a time-dependent covariate in a Cox model for each other toxicity, unadjusted for any other predictor. If the explanatory toxicity was censored before the dependent toxicity, the dependent toxicity was artificially censored at the same earlier time.

Genotype Imputation

Genetic data were imputed using, as reference haplotypes, the1000 Genomes Project Phase 3 (Haplotype release date October 2014) for chromosomes 1 to 22 and the 1000 Genomes Project Phase 1 (Haplotype ChrX release date Aug 2012) for chromosome X, since the phased data for Chr X from 1000GP Phase 3 was not available. A two-stage procedure used SHAPEIT (shapeit.v2.r790.Ubuntu_12.04.4.static) to derive phased genotypes (default parameters with the following modifications: 10 burn-in iterations, 10 pruning iterations, and 50 iterations to compute transition probabilities) and IMPUTEv2 (impute_v2.3.2_x86_64_static) to perform imputation of the phased data (default parameters with the following modifications: 5Mb non-overlapping intervals, 800 reference haplotypes to use as templates when imputing missing genotypes, and 500kb buffer region). 1000 Genomes Project variants whose minor allele frequency in Europeans and East Asians was lower than 0.001 were excluded from imputation. All OncoArray datasets were imputed jointly; the Affymetrix and Illumina CytoSNP12 datasets were imputed separately following the same procedure.

Fine-scale mapping

Genomic regions were defined as the 1Mb interval surrounding each statistically significant independent association. We re-imputed genotypes for the non-directly-genotyped variants using IMPUTE2 [15] and a reference panel [16] using the standard IMPUTE2 MCMC algorithm for follow-up imputation (see [17] for detailed description of the parameters used) to improve accuracy at low frequency variants. Variants with imputation info score ≥0.3 in all cohorts and MAF ≥0.02 in at least one cohort were included. 4,190 variants across the chr1:230337180-231337180 region; 3,776 at chr5:156903410_157903410 and 3,987 at chr9:30366808-31366808 were evaluated for hematuria, rectal bleeding or decreased urinary stream risk, respectively.

For each cohort, we ran grouped relative risk models independently and meta-analyzed the results, using a fixed-effects meta-analysis (*meta,* https://mathgen.stats.ox.ac.uk/genetics_software/meta/meta.html). Then, the most statistically significant variant (index variant at signal 1) was used to perform conditional analysis in each cohort independently. To define the cumulative posterior probability of the credible set, we estimated the empirical Bayes Factor [18].

The conditional results were meta-analyzed and the most significant variant (index variant at signal 2) selected. This loop continued until no variants at p-values of 10^{-4} remained at the region. A preliminary set of credible causal variants (CCVs) was then determined among the variants within two orders of magnitude from the index variant for each signal. The most significant variant (final index variant) within the set was identified by adjusting the effect of each signal by the additional signals. The final credible set was redefined among the variants with p-values within two orders of magnitude smaller than the index variant after being conditioned by the additional index variants at the region.

For each variant (i) we normalized its effect size ($\hat{\beta}_i$) and variance (σ_i) by its allele frequency (p_i) as follows

$$\beta_{Ni} = \hat{\beta}_i\sqrt{2p_i(1-p_i)}$$
$$\sigma_{Ni}^2 = \sigma_i^2 2p_i(1-p_i)$$

where p_i is the allele frequency for variant i in the OncoArray cohort, and estimated the prior variance (ω) using (Spencer et al., 2016) approach with normalized betas and normalized variance

$$\omega_N = \widehat{\beta_{N_{1\,30}}^{2}} - \sigma_{Nm}^2$$

We then estimated the cumulative posterior probability of the variants included in the credible set. For regions with more than one independent signal Bayes Factor was estimated using the summary statistics from the conditional analysis, after adjusting for other index variants at the region.

Credible causal variant (CCV) annotation

Variants were annotated with Variant Effect Predictor [19] to determine their effect on genes, transcripts, and protein sequences. To evaluate whether CCVs were located at regulatory regions, we overlapped our CCVs with Encode enhancer-like and promoter-like regions for 73 tissues and cells (primary, immortalized, *in vitro* differentiated) with available data for both enhancer- and promoter-like regions ([19-21]

and DCC accession: ENCSR037HRJ; GEO accession: GSE30567). In order to evaluate whether the CCVs could drive the expression of local genes, we accessed the GTEx Portal on 04/19/2018 to retrieve the metasoft results for all tissues in the V7 release. LocusZoom [22] was used to visualize associations for regions containing CCVs. Linkage disequilibrium was estimated using as reference the European ancestry populations from the 1000 Genomes Project (Phase 3, release 20130502; [16]).

Pathway Analysis

Gene- and pathway-based analysis was performed using *Pascal* (Pathway scoring algorithm) [23]. Gene-based scores were computed using the default "sum" option, which calculates the sum of chi-squared statistics and measures the strongest association signal per gene, respectively. SNPs were mapped to genes using a 100kb window surrounding each gene. Pathway-based scores are computed using a modified Fisher method, which improves statistical power compared with enrichment-based analysis while maintaining rigorous type I error control. The KEGG, Biocarta, and Reactome databases were queried for the pathway-based analysis.

Multivariable Modeling

Clinical variables were combined with genetic variants (identified via GWAS meta-analysis and from prior studies) using cohort-stratified grouped relative risk models, assuming an additive model for each variant, resulting in per allele hazard ratios (HR). Such grouped relative risk models estimate hazard ratios based on grouped survival data, assuming proportional hazards for the latent continuous survival times within each cohort stratum. The discrete monitoring times need not be equally spaced, nor need they be the same across cohort strata, but it is necessary that they be on the same temporal grid for all subjects within each cohort stratum. Confidence intervals and p-values were likelihood based and two-sided, with p-values ≤ 0.05 considered statistically significant.

Stepwise model selection was used to identify a parsimonious multivariable model for each toxicity outcome. For the multivariable cohort-stratified grouped relative risk models presented in Table 4, an alpha to enter of 0.10 and an alpha to stay of 0.05 were used as model selection parameters. Genetic variants were forced into the model in advance of the inclusion of any clinical variables. The large number of tied follow-up

times were handled using the exact method, equivalent to marginal likelihood (that Efron's method approximates) –not the discrete time method that assumes proportional odds rather than proportional hazards. The $\log_2$ transformation was used to symmetrize the distribution of strongly positively skewed continuous variables, thus reducing the influence of the most extreme observed covariate values and resulting in a hazard ratio per doubling of the predictor. The functional form of each continuous variable was chosen via model selection from the following options: linear; piecewise constant histospline with knots at one or more quartiles; or piecewise linear spline with knots at one or more quartiles, with the option to force the slope to be 0 to the left of the first knot (as a reference group, similar to that of a histospline).

Missing data were imputed within cohorts as follows. If a variable had ≤25% of values missing within a cohort, within-cohort mean imputation was used to impute the missing values. If a variable had >25% of values missing within a cohort, the variable was set to a constant of 0 within the cohort, allowing the hazard ratio for that variable to be estimated based only on cohorts with no more than 25% missing data, without requiring subjects missing data on a subset of variables within some cohorts to be excluded entirely from the analysis. This novel approach allowed us to at least partially adjust for variables that were available in some cohorts but not others, where the adjustment would be complete for variables that truly did not vary within cohorts in which they were missing –irrespective of the true constant value within each such cohort.

In addition to grouped relative risk regression, two other multivariable modeling methods were applied to derive separate predictive models for each of the four toxicity endpoints: Polygenic Risk Score (PRS) and a machine-learning method. In both methods, a "training" set was used to derive the model and a "test" set was used to evaluate model performance such that the training set was independent of the test set. The training set included a randomly selected 50% of the RAPPER study participants and all other cohorts; the testing set included the 50% of the RAPPER study participants not included in training data.

The first method, PRS, is a linear combination of risks of multiple SNPs identified by GWAS. The risk SNPs comprising the PRS were identified via GWAS meta-analysis of results from the cohorts comprising the training set for each of the four toxicity endpoints (rectal bleeding, increased urinary frequency, decreased urinary stream, and hematuria). On the training data, we tested several P_{meta} thresholds (1E-1, 1E-2, …, 1E-8) in selecting SNPs for PRS, followed by LD-pruned using 1000 Genomes Project EUR panel. Afterward, we

computed PRS for each individual of the testing data, and examine the association between PRS and toxicity endpoint.

The machine learning-based method, which was previously developed and applied to RNAseq-derived gene expression data [24], was used to derive multi-SNP models for predicting the toxicity outcomes considered in this study. Since this method was designed for binary classification tasks, we focused on the binarized versions of these endpoints (grade 2 or worse toxicity vs. grade 0 or 1 toxicity) in these experiments, as in the GWAS meta-analysis. We applied this method to the training set, with the constituent SNPs filtered at the same thresholds used for PGS, and evaluated the resultant models on the test set.

Data Management and Analysis

Genomic data were formatted using R (version 3.2.2, R Foundation for Statistical Computing, Vienna, Austria). Analysis was carried out using ProbABEL [25], which employs the coxfit2 function in the R package survival. GWAS results were meta-analyzed using Stata (version 14.2, StataCorp LLC, College Station, TX). Multivariable modeling was done using SAS (version 9.4, SAS Institute, Cary, NC). Pascal was used to compute gene and pathway scores [23].

References

1. Burnet NG, Elliott RM, Dunning A*, et al.* Radiosensitivity, radiogenomics and RAPPER. Clin Oncol (R Coll Radiol) 2006;18(7):525-8.

2. Sydes MR, Stephens RJ, Moore AR*, et al.* Implementing the UK Medical Research Council (MRC) RT01 trial (ISRCTN 47772397): methods and practicalities of a randomised controlled trial of conformal radiotherapy in men with localised prostate cancer. Radiother Oncol 2004;72(2):199-211.

3. Dearnaley D, Syndikus I, Mossop H*, et al.* Conventional versus hypofractionated high-dose intensity-modulated radiotherapy for prostate cancer: 5-year outcomes of the randomised, non-inferiority, phase 3 CHHiP trial. Lancet Oncol 2016;17(8):1047-60.

4. Dearnaley D, Syndikus I, Sumo G*, et al.* Conventional versus hypofractionated high-dose intensity-modulated radiotherapy for prostate cancer: preliminary safety results from the CHHiP randomised controlled trial. Lancet Oncol 2012;13(1):43-54.

5. Fachal L, Gomez-Caamano A, Barnett GC*, et al.* A three-stage genome-wide association study identifies a susceptibility locus for late radiotherapy toxicity at 2q24.1. Nat Genet 2014;46(8):891-4.

6. Ho AY, Atencio DP, Peters S*, et al.* Genetic predictors of adverse radiotherapy effects: the Gene-PARE project. Int J Radiat Oncol Biol Phys 2006;65(3):646-55.

7. Kerns SL, Stock R, Stone N*, et al.* A 2-stage genome-wide association study to identify single nucleotide polymorphisms associated with development of erectile dysfunction following radiation therapy for prostate cancer. Int J Radiat Oncol Biol Phys 2013;85(1):e21-8.

8. De Meerleer G, Vakaet L, Meersschout S*, et al.* Intensity-modulated radiotherapy as primary treatment for prostate cancer: acute toxicity in 114 patients. Int J Radiat Oncol Biol Phys 2004;60(3):777-87.

9. Kerns SL, Dorling L, Fachal L*, et al.* Meta-analysis of Genome Wide Association Studies Identifies Genetic Markers of Late Toxicity Following Radiotherapy for Prostate Cancer. EBioMedicine 2016;10:150-63.

10. LENT SOMA scales for all anatomic sites. Int J Radiat Oncol Biol Phys 1995;31(5):1049-91.

11. Dearnaley DP, Hall E, Lawrence D*, et al.* Phase III pilot study of dose escalation using conformal radiotherapy in prostate cancer: PSA control and side effects. Br J Cancer 2005;92(3):488-98.

12. *RTOG/EORTC Late Radiation Morbidity Scoring Schema*. https://www.rtog.org/ResearchAssociates/AdverseEventReporting/RTOGEORTCLateRadiationMorbidityScoringSchema.aspx.

13. National Cancer Institute. Common Terminology Criteria for Adverse Events v4.0. In. *NIH publication # 09-7473*; NCI, NIH, DHHS. May 29, 2009.

14. Barry MJ, Fowler FJ, Jr., O'Leary MP*, et al.* The American Urological Association symptom index for benign prostatic hyperplasia. The Measurement Committee of the American Urological Association. J Urol 1992;148(5):1549-57; discussion 1564.

15. Marchini J, Howie B, Myers S*, et al.* A new multipoint method for genome-wide association studies by imputation of genotypes. Nat Genet 2007;39(7):906-13.

16. Genomes Project C, Auton A, Brooks LD, *et al.* A global reference for human genetic variation. Nature 2015;526(7571):68-74.

17. Amos CI, Dennis J, Wang Z, *et al.* The OncoArray Consortium: A Network for Understanding the Genetic Architecture of Common Cancers. Cancer Epidemiol Biomarkers Prev 2017;26(1):126-135.

18. Wakefield J. A Bayesian measure of the probability of false discovery in genetic epidemiology studies. Am J Hum Genet 2007;81(2):208-27.

19. McLaren W, Gil L, Hunt SE, *et al.* The Ensembl Variant Effect Predictor. Genome Biol 2016;17(1):122.

20. Consortium EP. An integrated encyclopedia of DNA elements in the human genome. Nature 2012;489(7414):57-74.

21. Sloan CA, Chan ET, Davidson JM, *et al.* ENCODE data at the ENCODE portal. Nucleic Acids Res 2016;44(D1):D726-32.

22. Pruim RJ, Welch RP, Sanna S, *et al.* LocusZoom: regional visualization of genome-wide association scan results. Bioinformatics 2010;26(18):2336-7.

23. Lamparter D, Marbach D, Rueedi R, *et al.* Fast and Rigorous Computation of Gene and Pathway Scores from SNP-Based Summary Statistics. PLoS Comput Biol 2016;12(1):e1004714.

24. Pandey G, Pandey OP, Rogers AJ, *et al.* A Nasal Brush-based Classifier of Asthma Identified by Machine Learning Analysis of Nasal RNA Sequence Data. Sci Rep 2018;8(1):8826.

25. Aulchenko YS, Struchalin MV, van Duijn CM. ProbABEL package for genome-wide association analysis of imputed data. BMC Bioinformatics 2010;11:134.

26. Barnett GC, West CM, Coles CE, *et al.* Standardized Total Average Toxicity score: a scale- and grade-independent measure of late radiotherapy toxicity to facilitate pooling of data from different studies. Int J Radiat Oncol Biol Phys 2012;82(3):1065-74.

Supplementary Notes

The PRACTICAL Consortium (http://practical.icr.ac.uk/):

Rosalind A. Eeles[1,2], Brian E. Henderson[3*], Christopher A. Haiman[3], ZSofia Kote-Jarai[1], Fredrick R. Schumacher[4,5], Ali Amin Al Olama[6,7], Sara Benlloch[6,1], Kenneth Muir[8,9], Sonja I. Berndt[10], David V. Conti[3], Fredrik Wiklund[11], Stephen Chanock[10], Susan Gapstur[12], Victoria L. Stevens[12], Catherine M. Tangen[13], Jyotsna Batra[14,15], Judith Clements[14,15], Australian Prostate Cancer BioResource (APCB)[14], Henrik Gronberg[11], Nora Pashayan[16,17], Johanna Schleutker[18,19], Demetrius Albanes[10], Alicja Wolk[20, 21], Catharine West[22], Lorelei

Mucci[23], Géraldine Cancel-Tassin[24,25], Stella Koutros[10], Karina Dalsgaard Sorensen[26,27], Eli Marie Grindedal[28], David E. Neal[29,30,31], Freddie C. Hamdy[31], Jenny L. Donovan[32], Ruth C. Travis[33], Robert J. Hamilton[34], Sue Ann Ingles[3], Barry S. Rosenstein[35,36], Yong-Jie Lu[37], Graham G. Giles[38,39], Adam S. Kibel[40], Ana Vega[41], Manolis Kogevinas[42,43,44,45], Kathryn L. Penney[46], Jong Y. Park[47], Janet L. Stanford[48,49], Cezary Cybulski[50], Børge G. Nordestgaard[51,52], Hermann Brenner[53,54,55], Christiane Maier[56], Jeri Kim[57], Esther M. John[58,59], Manuel R. Teixeira[60,61], Susan L. Neuhausen[62], Kim De Ruyck[63], Azad Razack[64], Lisa F. Newcomb[48,65], Davor Lessel[66], Radka Kaneva[67], Nawaid Usmani[68,69], Frank Claessens[70], Paul A. Townsend[71], Manuela Gago-Dominguez[72,73], Monique J. Roobol[74], Florence Menegaux[75], Kay-Tee Khaw[76], Lisa Cannon-Albright[77,78], Hardev Pandha[79], Stephen N. Thibodeau[80]

[*]In memorium

[1] The Institute of Cancer Research, London, UK.
[2] Royal Marsden NHS Foundation Trust, London, UK.
[3] Department of Preventive Medicine, Keck School of Medicine, University of Southern California/Norris Comprehensive Cancer Center, Los Angeles, CA, USA.
[4] Department of Epidemiology and Biostatistics, Case Western Reserve University, Cleveland, OH, USA.
[5] Seidman Cancer Center, University Hospitals, Cleveland, OH, USA.
[6] Centre for Cancer Genetic Epidemiology, Department of Public Health and Primary Care, University of Cambridge, Strangeways Research Laboratory, Cambridge, UK.
[7] University of Cambridge, Department of Clinical Neurosciences, Cambridge, UK.
[8] Division of Population Health, Health Services Research and Primary Care, University of Manchester, Manchester, UK.
[9] Warwick Medical School, University of Warwick, Coventry, UK.
[10] Division of Cancer Epidemiology and Genetics, National Cancer Institute, NIH, Bethesda, MD, USA.
[11] Department of Medical Epidemiology and Biostatistics, Karolinska Institute, Stockholm, Sweden.
[12] Epidemiology Research Program, American Cancer Society, 250 Williams Street, Atlanta, GA, USA.
[13] SWOG Statistical Center, Fred Hutchinson Cancer Research Center, Seattle, WA, USA.
[14] Australian Prostate Cancer Research Centre-Qld, Institute of Health and Biomedical Innovation and School of Biomedical Science, Queensland University of Technology, Brisbane, Queensland, Australia.
[15] Translational Research Institute, Brisbane, Queensland, Australia.
[16] University College London, Department of Applied Health Research, London, UK.
[17] Centre for Cancer Genetic Epidemiology, Department of Oncology, University of Cambridge, Strangeways Laboratory, Cambridge, UK.
[18] Department of Medical Biochemistry and Genetics, Institute of Biomedicine, University of Turku, Finland.
[19] Tyks Microbiology and Genetics, Department of Medical Genetics, Turku University Hospital, Finland.
[20] Division of Nutritional Epidemiology, Institute of Environmental Medicine, Karolinska Institutet, Sweden.
[21] Department of Surgical Sciences, Uppsala University, Uppsala, Sweden.
[22] Division of Cancer Sciences, University of Manchester, Manchester Academic Health Science Centre, Radiotherapy Related Research, Manchester NIHR Biomedical Research Centre, The Christie Hospital NHS Foundation Trust, Manchester, UK.
[23] Department of Epidemiology, Harvard T.H Chan School of Public Health, Boston, MA, USA.
[24] CeRePP, Tenon Hospital, Paris, France.
[25] UPMC Sorbonne Universites, GRC N°5 ONCOTYPE-URO, Tenon Hospital, Paris, France.
[26] Department of Molecular Medicine, Aarhus University Hospital, Denmark.
[27] Department of Clinical Medicine, Aarhus University, Denmark.
[28] Department of Medical Genetics, Oslo University Hospital, Norway.
[29] University of Cambridge, Department of Oncology, Addenbrooke's Hospital, Cambridge, UK.
[30] Cancer Research UK Cambridge Research Institute, Li Ka Shing Centre, Cambridge, UK.
[31] Nuffield Department of Surgical Sciences, University of Oxford, Oxford, UK, Faculty of Medical Science, University of Oxford, John Radcliffe Hospital, Oxford, UK.
[32] School of Social and Community Medicine, University of Bristol, Bristol, UK.
[33] Cancer Epidemiology Unit, Nuffield Department of Population Health University of Oxford, Oxford, UK.
[34] Dept. of Surgical Oncology, Princess Margaret Cancer Centre, Toronto, Canada.
[35] Department of Radiation Oncology, Icahn School of Medicine at Mount Sinai, New York, NY, USA.

[36] Department of Genetics and Genomic Sciences, Icahn School of Medicine at Mount Sinai, New York, NY, USA.
[37] Centre for Molecular Oncology, Barts Cancer Institute, Queen Mary University of London, John Vane Science Centre, London, UK.
[38] Cancer Epidemiology & Intelligence Division, The Cancer Council Victoria, Melbourne, Victoria, Australia.
[39] Centre for Epidemiology and Biostatistics, Melbourne School of Population and Global Health, The University of Melbourne, Melbourne, Australia.
[40] Division of Urologic Surgery, Brigham and Womens Hospital, Boston, MA, USA.
[41] Fundación Pública Galega de Medicina Xenómica-SERGAS, Grupo de Medicina Xenómica, CIBERER, IDIS, Santiago de Compostela, Spain.
[42] Centre for Research in Environmental Epidemiology (CREAL), Barcelona Institute for Global Health (ISGlobal), Barcelona, Spain.
[43] CIBER Epidemiología y Salud Pública (CIBERESP), Madrid, Spain.
[44] IMIM (Hospital del Mar Research Institute), Barcelona, Spain.
[45] Universitat Pompeu Fabra (UPF), Barcelona, Spain.
[46] Channing Division of Network Medicine, Department of Medicine, Brigham and Women's Hospital/Harvard Medical School, Boston, MA, USA.
[47] Department of Cancer Epidemiology, Moffitt Cancer Center, Tampa, USA.
[48] Division of Public Health Sciences, Fred Hutchinson Cancer Research Center, Seattle, Washington, USA.
[49] Department of Epidemiology, School of Public Health, University of Washington, Seattle, Washington, USA.
[50] International Hereditary Cancer Center, Department of Genetics and Pathology, Pomeranian Medical University, Szczecin, Poland.
[51] Faculty of Health and Medical Sciences, University of Copenhagen, Denmark.
[52] Department of Clinical Biochemistry, Herlev and Gentofte Hospital, Copenhagen University Hospital, Herlev, Denmark.
[53] Division of Clinical Epidemiology and Aging Research, German Cancer Research Center (DKFZ), Heidelberg, Germany.
[54] German Cancer Consortium (DKTK), German Cancer Research Center (DKFZ), Heidelberg, Germany.
[55] Division of Preventive Oncology, German Cancer Research Center (DKFZ) and National Center for Tumor Diseases (NCT), Heidelberg, Germany.
[56] Institute for Human Genetics, University Hospital Ulm, Ulm, Germany.
[57] The University of Texas MD Anderson Cancer Center, Department of Genitourinary Medical Oncology, Houston, TX, USA.
[58] Cancer Prevention Institute of California, Fremont, CA, USA.
[59] Department of Health Research & Policy (Epidemiology) and Stanford Cancer Institute, Stanford University School of Medicine, Stanford, CA , USA.
[60] Department of Genetics, Portuguese Oncology Institute of Porto, Porto, Portugal.
[61] Biomedical Sciences Institute (ICBAS), University of Porto, Porto, Portugal.
[62] Department of Population Sciences, Beckman Research Institute of the City of Hope, Duarte, CA, USA.
[63] Ghent University, Faculty of Medicine and Health Sciences, Basic Medical Sciences, Gent, Belgium.
[64] Department of Surgery, Faculty of Medicine, University of Malaya, Kuala Lumpur, Malaysia.
[65] Department of Urology, University of Washington, Seattle, WA, USA.
[66] Institute of Human Genetics, University Medical Center Hamburg-Eppendorf, Hamburg, Germany.
[67] Molecular Medicine Center, Department of Medical Chemistry and Biochemistry, Medical University, Sofia, Bulgaria.
[68] Department of Oncology, Cross Cancer Institute, University of Alberta, Edmonton, Alberta, Canada.
[69] Division of Radiation Oncology, Cross Cancer Institute, Edmonton, Alberta, Canada.
[70] Molecular Endocrinology Laboratory, Department of Cellular and Molecular Medicine, KU Leuven, Leuven, Belgium.
[71] Institute of Cancer Sciences, Manchester Cancer Research Centre, University of Manchester, Manchester Academic Health Science Centre, St Mary's Hospital, Manchester, UK.
[72] Genomic Medicine Group, Galician Foundation of Genomic Medicine, Instituto de Investigacion Sanitaria de Santiago de Compostela (IDIS), Complejo Hospitalario Universitario de Santiago, Servicio Galego de Saúde, SERGAS, Santiago De Compostela, Spain.
[73] University of California San Diego, Moores Cancer Center, La Jolla, CA, USA.
[74] Department of Urology, Erasmus University Medical Center, Rotterdam, the Netherlands.

[75] Cancer & Environment Group, Center for Research in Epidemiology and Population Health (CESP), INSERM, University Paris-Sud, University Paris-Saclay, Villejuif, France.
[76] Clinical Gerontology Unit, University of Cambridge, Cambridge, UK.
[77] Division of Genetic Epidemiology, Department of Medicine, University of Utah School of Medicine, Salt Lake City, Utah, USA.
[78] George E. Wahlen Department of Veterans Affairs Medical Center, Salt Lake City, UT, USA.
[79] The University of Surrey, Guildford, Surrey, UK.
[80] Department of Laboratory Medicine and Pathology, Mayo Clinic, Rochester, MN, USA.

Supplementary Tables

Supplementary Table 1. Definitions of toxicity grades. Semicolons indicate “or”.

Toxicity endpoint by Study and toxicity grading tool	Grade definitions
Increased urinary frequency[*]	
RAPPER<br>LENT-SOMA subjective [LS-s] and management [LS-m] scales; Royal Marsden Hospital [RMH] scale	0 = daytime frequency >4 hour intervals (LS-s) & no treatment (LS-m) & nocturia 0-1 times (RMH)<br>1 = daytime frequency 3-4 hour intervals (LS-s) or 2-3 hour intervals (LS-s); alkalization (LS-m); nocturia 2-3 times (RMH)<br>2 = daytime frequency 1-2 hour intervals (LS-s); anti-spasmotic (LS-m) OR regular narcotic (LS-m); nocturia 4-5 times (RMH)<br>3 = daytime frequency hourly (LS-s); nocturia 6-8 times (RMH) or >8 times (RMH)<br>4 = cystectomy (LS-m)
RADIOGEN<br>CCI-EBRT<br>UGhent[†]<br>PRRG<br>CTCAEv3.0 - Urinary frequency/urgency	0 = No toxicity<br>1 = Increase in frequency or nocturia up to 2 x normal; enuresis<br>2 = Frequency or nocturia >2 x normal but <hourly<br>3 = Frequency or nocturia ≥1 x/hr; urgency; catheter indicated
Gene-PARE<br>NTMC<br>American Urological Association Symptom Score Q2[‡] and Q7[§]	0 = Had to urinate again less than 2 hours after you have urinated ‘not at all’ or ‘less than 1 time in 5’ & no nocturia or nocturia 1 time<br>1 = Had to urinate again less than 2 hours after you have urinated ‘less than ½ the time’ or ‘about ½ the time’; nocturia 2 times or 3 times<br>2 = Had to urinate again less than 2 hours after you have urinated ‘more than ½ the time’; nocturia 4 times or 5 times<br>3 = Had to urinate again less than 2 hours after you have urinated ‘more than ½ the time’ ‘almost always’
Decreased urinary stream[‖]	
RAPPER<br>LENT-SOMA subjective [LS-s] and management [LS-m] scales	0 = No toxicity (LS-s) & no treatment for decreased stream (LS-m)<br>1 = Occasionally weak (LS-s)<br>2 = Intermittent (LS-s); < 1/day self-catheterization (LS-m)<br>3 = Persistent but incomplete obstruction (LS-s); dilation or > 1/day self-catheterization (LS-m)<br>4 = complete obstruction (LS-s); permanent catheter or surgical intervention (LS-m)
RADIOGEN<br>CCI-EBRT<br>PRRG<br>UGhent<br>CTCAEv3.0 - Urinary retention	0 = No toxicity<br>1 = Hesitancy or dribbling, no significant residual urine; retention occurring during the immediate postoperative period<br>2 = Hesitancy requiring medication; or operative bladder atony requiring indwelling catheter beyond immediate postoperative period but for <6 weeks<br>3 = More than daily catheterization indicated; urological intervention indicated (e.g., TURP, suprapubic tube, urethrotomy)

	4 = Life-threatening consequences; organ failure (e.g., bladder rupture); operative intervention requiring organ resection indicated
Gene-PARE<br>NTMC<br>American Urological Association Symptom Score Q5[¶]	0 = Had a weak urinary stream ‘not at all’ or ‘less than 1 time in 5’<br>1 = Had a weak urinary stream ‘less than ½ the time’<br>2 = Had a weak urinary stream ‘about ½ the time’ or ‘more than ½ the time’<br>3 = Had a weak urinary stream ‘almost always’
Hematuria[#]	
RAPPER<br>LENT-SOMA subjective [LS-s], objective [LS-o], and management [LS-m] scales; RTOG late effects [RTOG] scale	0 = No toxicity (LS-s) & no treatment for hematuria (LS-m) & no toxicity (RTOG)<br>1 = Microscopic, normal hemoglobin (LS-o)<br>2 = Occasional or Intermittent (LS-s); Iron therapy or Occasional transfusion/single cauterization (LS-m); Intermittent macroscopic, < 10% decrease in hemoglobin (LS-o); (RTOG)<br>3 = Persistent with clot (LS-s); Persistent macroscopic, 10-20% decrease in hemoglobin (LS-o); Frequent transfusion or coagulation (LS-m); (RTOG)<br>4 = Refractory (LS-s); Surgical intervention (LS-m); > 20% decrease in hemoglobin (LS-o); (RTOG)
RADIOGEN<br>CCI-EBRT<br>PRRG<br>CTCAEv3.0 - Cystitis	0 = None<br>1 = Asymptomatic<br>2 = Frequency with dysuria; macroscopic hematuria<br>3 = Transfusion; IV pain medications; bladder irrigation indicated<br>4 = Catastrophic bleeding; major non-elective intervention indicated
Gene-PARE<br>Institutional scale	0 = None or microscopic hematuria<br>2 = Macroscopic hematuria
Rectal bleeding[**]	
RAPPER<br>LENT-SOMA objective (LS-o) and management (LS-m) scales; Royal Marsden Hospital (RMH) scale	0 = None or Occult (LS-o) & None (LS-m) & None (RMH)<br>1 = Stool softener, iron therapy (LS-m); Occasional-no treatment (RMH)<br>2 = Occasionally, >2x/week (LS-o); Occasional transfusion (LS-m); Moderate-simple OPD treatment (RMH)<br>3 = Severe (blood transfusion, surgery)<br>4 = Life-threatening consequences; major urgent intervention indicated
RADIOGEN<br>CCI-EBRT<br>CTCAEv3.0 - GI hemorrhage	0 = None<br>1 = Mild, intervention (other than iron supplements) not indicated<br>2 = Symptomatic and medical intervention or minor cauterization indicated<br>3 = Transfusion, interventional radiology, endoscopic, or operative intervention indicated; radiation therapy (i.e., haemostasis of bleeding site)<br>4 = Life-threatening consequences; major urgent intervention indicated
UGhent<br>CCI-BT<br>Institutional scale	0 = None<br>2 = Bleeding present

[*] Increased urinary frequency was not analyzed in CCI-BT because pre-radiotherapy assessments were more than one year prior to starting radiotherapy for the majority of participants. Abbreviations: OPD, outpatient department; NA, grade not applicable

[†] UGhent used an institutional-specific scaled based on CTCAEv3.0

[‡] During the past month or so, how often have you had to urinate again less than two hours after you finished urinating?

[§] Over the past month, how many times per night did you most typically get up to urinate from the time you went to bed at night until the time you got up in the morning?
[||] Decreased urinary stream was not analyzed in CCI-BT because pre-radiotherapy assessments were more than one year prior to starting radiotherapy for the majority of participants.
[¶] During the past month, how often have you had a weak urinary stream?
[#] Endpoint not available in CCI-BT, UGhent or NTMC
[**] Rectal bleeding was not assessed in NTMC or PRRG. Rectal bleeding was assigned a single grade in GenePARE using information across all follow up assessments, and so this endpoint was not available for analysis using Cox proportional hazards modeling.

Supplementary Table 2. Power* to detect statistically significant associations among 3,871 radiotherapy patients

Per-allele Odds Ratio	Minor allele frequency, %					
	**0.05**	**0.10**	**0.15**	**0.25**	**0.35**	**0.50**
1.15	0	0	0	0	0	0
1.25	0	0	0	0	1	1
1.50	0	2	7	26	43	52
1.75	3	22	54	89	97	99
2.00	12	65	93	100	100	100
2.25	34	93	100	100	100	100

* Assuming a type I error rate of $5x10^{-8}$ and number of toxicity cases and non-toxicity controls for the most rare toxicity, hematuria, included in the study. Power for more prevalent toxicities is greater than that presented in the table.

Supplementary Table 3. Top SNPs that did not reach genome-wide significance and rare variants

rsID	location (chr_position_a1_a2)	Toxicity Outcome	MAF*	Info†	HR‡ (95% CI)	P_{meta}§	BFDP‖
Common SNPs with $5x10^{-8}<P_{meta}<5x10^{-7}$							
rs9644474	8_137163144_C_T	Rectal bleeding	0.05	0.78	2.15 (1.64, 2.81)¶	$7.9x10^{-08}$	9.1
rs75759941	23_38151042_T_C	STAT score	0.05	0.99	0.25 (0.16, 0.34) #	$9.3x10^{-8}$	5.8
rs11122572	1_230825427_T_G	Hematuria	0.09	0.99	1.86 (1.48, 2.34)	$9.8x10^{-08}$	8.2
rs368141164	11_116445686_A_T	Increased urinary frequency	0.06	0.95	1.87 (1.48, 2.36)	$1.8x10^{-07}$	13.8
NA	18_57916552_A_C	STAT score	0.26	1.00	0.07 (0.04, 0.10) #	$1.6x10^{-7}$	23.5
rs2031925	10_30680024_T_C	Rectal bleeding	0.05	0.59	2.43 (1.73, 3.40)	$2.4x10^{-07}$	33.1
rs17190422	2_56856260_A_G	Hematuria	0.06	0.90	2.57 (1.79, 3.68)	$3.1x10^{-07}$	43.0
rs74346764	3_145230069_G_A	Rectal bleeding	0.05	0.89	2.52 (1.77, 3.58)	$3.1x10^{-07}$	41.2
rs72993079	19_6573511_C_T	Hematuria	0.05	0.75	2.14 (1.60, 2.87)	$3.5x10^{-07}$	30.3
rs9832989	3_133701823_A_G	Hematuria	0.06	0.83	4.45 (2.50, 7.92)	$3.8x10^{-07}$	88.9
rs60424486	7_15410311_G_INS	Decreased urinary stream	0.08	0.93	1.84 (1.45, 2.33)	$4.0x10^{-07}$	24.1
rs11624322	14_37058609_C_T	Decreased urinary stream	0.37	0.99	1.51 (1.29, 1.77)	$4.2x10^{-07}$	19.0
rs61871726	10_122293568_T_C	Rectal bleeding	0.07	0.92	1.98 (1.52, 2.59)	$4.8x10^{-07}$	31.2
rs2237706	7_107633171_C_T	Rectal bleeding	0.09	0.68	1.94 (1.50, 2.52)	$4.8x10^{-07}$	30.0
rs6791846	3_177119317_G_A	Increased urinary frequency	0.45	0.90	1.47 (1.26, 1.71)	$5.0x10^{-07}$	21.3
Rare variants with $P_{meta}<5x10^{-7}$							
NA	12_102080173_A_G	RecBld	0.03	0.82	3.57 (2.37, 5.39)	$1.4x10^{-09}$	2.6
rs180958289	19_1543771_G_A	DecStrm	0.02	0.65	7.78 (3.92, 15.5)	$4.6x10^{-09}$	72.4
rs13403657	2_241943450_A_G	Hematuria	0.03	0.82	3.26 (2.17, 4.90)	$1.4x10^{-08}$	11.8
rs139239158	3_59904329_C_G	DecStrm	0.02	0.80	3.69 (2.32, 5.87)	$3.4x10^{-08}$	34.0
rs191705561	4_184691564_G_T	UrineFreq	0.01	0.54	3.05 (2.05, 4.53)	$3.7x10^{-08}$	18.9
rs148048756	8_562963_A_G	RecBld	0.03	0.78	4.83 (2.75, 8.47)	$4.0x10^{-08}$	65.7
rs75988504	2_141730052_G_A	DecStrm	0.04	0.99	2.01 (1.56, 2.58)	$4.9x10^{-08}$	5.5
rs139288166	8_705922_A_C	RecBld	0.03	0.85	4.30 (2.53, 7.31)	$6.9x10^{-08}$	64.4
rs139882217	3_54729912_C_T	Hematuria	0.04	0.74	4.78 (2.71, 8.44)	$6.9x10^{-08}$	73.8
NA	12_31100664_C_G	Hematuria	0.02	0.99	9.22 (4.06, 20.9)	$1.1x10^{-07}$	97.5
NA	12_33380941_C_T	STAT score	0.04	0.99	0.17 (0.11, 0.23) #	$1.1x10^{-07}$	8.0
rs112193369	1_7558251_A_INS	DecStrm	0.02	0.91	4.09 (2.43, 6.90)	$1.2x10^{-07}$	70.3
rs4688181	3_63989456_A_G	Hematuria	0.04	0.92	3.22 (2.09, 4.98)	$1.3x10^{-07}$	47.3
rs149176864	8_30559235_G_A	Rectal bleeding	0.02	0.73	2.99 (1.99, 4.49)	$1.4x10^{-07}$	41.4
rs3739643	9_4600633_C_T	Hematuria	0.04	0.89	3.44 (2.17, 5.47)	$1.7x10^{-07}$	60.3
rs73539559	6_112344737_C_T	Hematuria	0.02	0.85	2.86 (1.93, 4.26)	$2.0x10^{-07}$	44.4
rs190601686	9_86200809_C_T	Hematuria	0.01	0.79	9.13 (3.95, 21.1)	$2.3x10^{-07}$	98.3
rs149927798	3_26548947_C_T	RecBld	0.02	0.55	3.98 (2.36, 6.71)	$2.3x10^{-07}$	77.8
rs490393	6_114228700_C_A	Hematuria	0.03	0.82	2.96 (1.96, 4.47)	$2.4x10^{-07}$	51.6
rs73712257	8_138319386_A_T	RecBld	0.03	0.49	4.72 (2.62, 8.50)	$2.4x10^{-07}$	87.6
rs144214859	2_68624610_G_A	Hematuria	0.02	0.99	2.58 (1.80, 3.70)	$2.5x10^{-07}$	39.3
rs61415111	11_17420841_A_C	UrineFreq	0.02	0.61	4.60 (2.57, 8.24)	$3.0x10^{-07}$	88.3
rs77581414	19_10934094_T_A	DecStrm	0.04	0.93	6.07 (3.04, 12.1)	$3.1x10^{-07}$	95.5
rs76661052	5_144759200_C_T	Hematuria	0.03	0.86	2.33 (1.68, 3.22)	$3.3x10^{-07}$	35.5
rs78166464	19_5200653_C_T	DecStrm	0.01	0.95	2.83 (1.90, 4.21)	$3.4x10^{-07}$	54.5
rs186353960	4_123920497_G_A	UrineFreq	0.04	0.66	2.44 (1.73, 3.44)	$3.4x10^{-07}$	40.5

rs11687040	2_238080051_G_A	DecStrm	0.01	0.99	3.37 (2.11, 5.40)	$4.2x10^{-07}$	74.5
rs73046248	3_21759604_T_C	DecStrm	0.04	0.85	2.40 (1.71, 3.36)	$4.2x10^{-07}$	43.3
rs192744896	15_27746339_T_C	RecBld	0.03	0.57	3.72 (2.23, 6.18)	$4.3x10^{-07}$	81.5
rs141203061	3_36244696_A_T	UrineFreq	0.03	0.86	2.71 (1.84, 4.00)	$4.3x10^{-07}$	55.7
rs113370662	4_3537697_C_T	DecStrm	0.04	0.81	2.78 (1.87, 4.15)	$4.5x10^{-07}$	59.3
rs138731641	9_83229027_GA_G	Hematuria	0.04	0.92	5.46 (2.82, 10.6)	$4.8x10^{-07}$	95.3
rs72915971	11_56925541_C_G	RecBld	0.02	0.95	2.16 (1.60, 2.91)	$5.0x10^{-07}$	38.1

[*] Minor allele frequency is from PRACTICAL oncoarray samples of European ancestry. Abbreviations: SNP, single nucleotide polymorphism; MAF, minor allele frequency; HR, hazard ratio; CI, confidence interval; BFDP, Bayesian false discovery probability; NA, not available; INS, insertion.

[†] Imputation info score values are from the oncoarray.

[‡] Hazard ratio corresponds to the minor allele with the major allele treated as the reference group.

[§] Two-sided P_{meta} was calculated using a Wald test.

[||] BFPD estimated assuming a prior variance, W = 0.32^2, and prior probability of a non-null association 0.0001

[¶] Hazard ratio is for the major allele with the minor allele treated as the reference group

[#] Beta coefficient from linear regression of STAT score at 2 years after radiotherapy

Supplementary Table 4. Credible causal variants identified by fine-scale mapping.

rsid	Position*	Ref Allele	Effect Allele	Info†	Info‡	Info§	Info‖	CCI-BT EAF	Gene PARE-II EAF	RADIO-GEN EAF	RAPPER-II EAF	UGhent EAF	CCI-ERBT EAF	Gene PARE-I EAF	RAPPER-I EAF
chr1:230337180-231337180, associated with hematuria															
Signal 1															
rs11122572	230825427	T	G	0.96	0.96	0.95	0.93	--	0.09	0.10	0.08	0.09	0.09	0.07	0.09
rs4846866	230836065	T	C	1.00	0.99	0.97	0.92	--	0.06	0.09	0.07	0.07	0.08	0.06	0.08
rs61762468	230836281	G	C	0.98	0.96	0.98	0.90	--	0.06	0.09	0.07	0.08	0.08	0.06	0.08
rs56117713	230836568	T	C	0.99	1.00	0.99	0.92	--	0.06	0.08	0.07	0.07	0.08	0.06	0.08
rs16852352	230836786	T	G	0.99	1.00	1.00	0.92	--	0.06	0.08	0.07	0.07	0.08	0.06	0.08
rs11122573	230837180	C	T	0.99	1.00	1.00	0.92	--	0.06	0.08	0.07	0.07	0.08	0.06	0.08
rs10864770	230837437	G	A	0.99	0.97	1.00	0.92	--	0.06	0.08	0.07	0.07	0.08	0.06	0.08
rs10864771	230837672	T	G	0.99	1.00	0.99	0.92	--	0.06	0.08	0.07	0.07	0.08	0.06	0.08
rs11122574	230837808	C	T	0.97	0.96	0.96	0.86	--	0.04	0.06	0.05	0.05	0.07	0.04	0.05
rs1926723	230840096	T	C	0.98	0.97	1.00	0.92	--	0.06	0.08	0.07	0.07	0.08	0.06	0.08
rs1926722	230840197	C	A	0.98	1.00	1.00	0.92	--	0.06	0.08	0.07	0.07	0.08	0.06	0.08
rs11122575	230840269	A	G	0.98	1.00	1.00	0.92	--	0.06	0.08	0.07	0.07	0.08	0.06	0.08
rs11568056	230842497	C	T	0.97	0.98	0.96	0.91	--	0.06	0.09	0.07	0.08	0.08	0.06	0.08
rs11122576	230846679	T	C	1.00	1.00	0.98	0.95	--	0.06	0.09	0.07	0.08	0.08	0.06	0.08
rs11568028	230847244	C	T	1.00	1.00	1.00	0.95	--	0.06	0.09	0.07	0.08	0.08	0.06	0.08
rs11122578	230847789	G	A	1.00	1.00	1.00	0.95	--	0.06	0.09	0.07	0.08	0.08	0.06	0.08
rs3789679	230849694	G	A	0.98	0.99	0.97	0.94	--	0.06	0.09	0.07	0.08	0.08	0.06	0.08
rs9804147	230853359	G	A	0.99	0.99	0.99	0.98	--	0.10	0.11	0.10	0.10	0.09	0.09	0.10
rs9804153	230853905	G	T	0.99	1.00	0.99	0.98	--	0.10	0.11	0.10	0.10	0.09	0.09	0.10
rs4028824	230854141	G	A	0.99	1.00	1.00	0.98	--	0.10	0.11	0.10	0.10	0.09	0.09	0.10
rs10864773	230856996	C	T	0.99	1.00	0.97	0.97	--	0.06	0.09	0.09	0.09	0.09	0.07	0.09
rs61762467	230836254-5	-	T	0.99	1.00	0.99	0.92	--	0.06	0.08	0.07	0.07	0.08	0.06	0.08
Signal 2															
rs4846857	230436175	A	G	1.00	0.64	0.61	0.48	--	0.86	0.87	0.85	0.85	0.86	0.84	0.85
rs79434380	230451274	C	T	0.68	0.52	0.57	0.45	--	0.03	0.02	0.01	0.01	0.02	0.03	0.02
rs564325629	230451573	G	-	0.71	0.52	0.53	0.45	--	0.03	0.01	0.01	0.01	0.01	0.03	0.01
rs147121532	230451849	T	C	0.72	0.52	0.53	0.45	--	0.03	0.01	0.01	0.01	0.01	0.02	0.01
rs28605378	230882070	G	A	0.96	0.95	0.78	0.89	--	0.08	0.07	0.06	0.08	0.06	0.09	0.06
rs12095859	230882213	T	C	0.95	0.95	0.78	0.90	--	0.08	0.07	0.06	0.07	0.06	0.09	0.06
rs12091328	230884209	G	C	0.99	0.96	0.80	0.90	--	0.08	0.07	0.06	0.08	0.06	0.09	0.06
rs12059171	230885399	T	G	0.99	0.96	0.81	0.91	--	0.08	0.07	0.06	0.07	0.06	0.09	0.06
rs12060898	230887539	A	G	0.98	0.96	0.82	0.90	--	0.08	0.07	0.06	0.07	0.06	0.09	0.06
rs75991123	230892946	C	A	0.96	0.97	0.89	0.94	--	0.06	0.06	0.05	0.06	0.05	0.08	0.06

chr5:156903410-157903410, associated with rectal bleeding															
rs17055178	157403410	A	G	0.96	0.94	--	0.96	0.08	--	0.08	0.08	0.07	0.07	--	0.06
rs13180537	157419681	C	T	0.90	0.81	--	0.88	0.07	--	0.07	0.07	0.06	0.05	--	0.05
rs78394554	157438561	A	C	0.93	0.91	--	0.94	0.08	--	0.08	0.08	0.07	0.06	--	0.06
rs34395161	157440433	C	A	0.90	0.80	--	0.89	0.07	--	0.07	0.07	0.06	0.05	--	0.05
rs35327501	157452625	G	A	0.94	0.91	--	0.94	0.08	--	0.08	0.08	0.07	0.06	--	0.06
rs4704767	157470956	C	T	1.00	1.00	--	0.98	0.08	--	0.11	0.10	0.09	0.08	--	0.08
rs35929592	157471129	C	T	1.00	1.00	--	0.98	0.08	--	0.11	0.10	0.09	0.08	--	0.08
rs35766682	157472745	C	T	0.99	1.00	--	0.98	0.08	--	0.11	0.10	0.09	0.09	--	0.08
rs10515757	157473330	A	T	1.00	1.00	--	0.98	0.08	--	0.11	0.10	0.09	0.08	--	0.08
rs35153425	157478391	A	G	1.00	1.00	--	0.98	0.08	--	0.11	0.10	0.09	0.08	--	0.08
rs17055241	157480829	G	A	1.00	1.00	--	0.98	0.08	--	0.11	0.10	0.09	0.08	--	0.08
rs1040926	157483536	A	G	1.00	1.00	--	0.98	0.09	--	0.11	0.10	0.09	0.08	--	0.08
rs13179825	157485371	C	T	1.00	1.00	--	0.98	0.08	--	0.11	0.10	0.08	0.08	--	0.08
rs13184115	157485718	C	A	1.00	1.00	--	0.97	0.09	--	0.11	0.10	0.08	0.09	--	0.08
rs17229231	157486935	C	T	0.99	1.00	--	0.98	0.08	--	0.11	0.10	0.09	0.08	--	0.08
chr9:30366808-31366808, associated with decreased urinary stream															
rs10969913	30866808	A	G	0.95	0.83	0.98	0.70	--	0.03	0.01	0.01	--	0.02	0.04	0.01
rs7868409	30868163	T	C	0.95	1.00	0.96	0.72	--	0.03	0.01	0.01	--	0.02	0.04	0.01
rs10969915	30868871	A	C	0.95	0.88	0.98	0.72	--	0.03	0.01	0.01	--	0.01	0.04	0.01
rs10969916	30869372	C	G	0.95	0.86	0.98	0.72	--	0.03	0.01	0.01	--	0.01	0.04	0.01
rs1412406	30869687	C	T	0.95	0.88	0.98	0.71	--	0.03	0.01	0.01	--	0.01	0.04	0.01
rs539024322	30873589	A	AT	0.94	0.87	0.97	0.69	--	0.03	0.01	0.01	--	0.02	0.04	0.02
rs10969918	30875780	T	C	0.97	1.00	0.95	0.71	--	0.03	0.01	0.01	--	0.02	0.04	0.01
rs112134389	30876262	T	C	0.97	0.92	0.99	0.71	--	0.03	0.01	0.01	--	0.02	0.04	0.01
rs111692482	30876567	C	T	0.97	0.93	0.99	0.71	--	0.03	0.01	0.01	--	0.02	0.04	0.01
rs10969920	30876943	A	C	0.97	1.00	1.00	0.71	--	0.03	0.01	0.01	--	0.02	0.04	0.01
rs10969923	30877317	T	C	0.96	0.88	0.97	0.68	--	0.03	0.01	0.01	--	0.02	0.04	0.02
rs73644367	30877456	A	C	0.98	0.92	0.98	0.71	--	0.03	0.01	0.01	--	0.02	0.04	0.01
rs73644368	30877580	A	G	0.97	0.91	0.97	0.71	--	0.03	0.01	0.01	--	0.02	0.04	0.01
rs10969926	30878999	T	A	0.98	1.00	1.00	0.71	--	0.03	0.01	0.01	--	0.02	0.04	0.01
rs77191587	30866630-2	ATT	-	0.95	0.87	0.98	0.71	--	0.03	0.01	0.01	--	0.01	0.04	0.01

* GRCh37/hg19, bp; Abbreviations: EAF, effect allele frequency; -- indicates that a value was not available.

† Info score from imputation of SNP data generated via the Illumina Oncoarray

‡ Info score from imputation of SNP data generated in CCI-EBRT via the AffySNPv6.0 array

§ Info score from imputation of SNP data generated in GenePARE-I via the AffySNPv6.0 array

|| Info score from imputation of SNP data generated in RAPPER-I via the Illumina CytoSNP12 array

Supplementary Table 5. Association results for credible causal variants identified by fine-scale mapping

rsid	Non-conditional results*					Conditional results				
	HR (95% CI)	*P*†	Q	I^2	P_{het}‡	HR (95% CI)	*P*†	Q	I^2	P_{het}‡
chr1:230337180-231337180, associated with hematuria										
Signal 1										
rs11122572	1.85 (1.47-2.32)	1.9E-07	8.0	24.7	0.24	1.82 (1.44-2.29)	3.9E-07	8.5	29.2	0.20
rs4846866	1.90 (1.50-2.40)	1.2E-07	8.5	29.0	0.21	1.87 (1.48-2.38)	2.4E-07	9.7	37.8	0.14
rs61762468	1.90 (1.50-2.41)	1.3E-07	8.1	25.9	0.23	1.87 (1.47-2.38)	2.8E-07	9.2	34.8	0.16
rs56117713	1.89 (1.49-2.40)	1.8E-07	7.8	23.6	0.25	1.86 (1.47-2.37)	3.7E-07	9.0	33.1	0.18
rs16852352	1.89 (1.49-2.40)	1.9E-07	7.9	23.9	0.25	1.86 (1.46-2.37)	3.9E-07	9.0	33.3	0.17
rs11122573	1.89 (1.49-2.40)	1.9E-07	7.8	23.2	0.25	1.86 (1.46-2.37)	3.9E-07	8.9	32.8	0.18
rs10864770	1.89 (1.49-2.40)	1.8E-07	7.8	22.8	0.26	1.86 (1.47-2.37)	3.7E-07	8.9	32.5	0.18
rs10864771	1.89 (1.49-2.40)	1.8E-07	7.8	22.8	0.26	1.86 (1.47-2.37)	3.7E-07	8.9	32.5	0.18
rs11122574	1.92 (1.43-2.57)	1.1E-05	9.3	35.7	0.16	1.90 (1.42-2.55)	1.6E-05	10.5	42.7	0.11
rs1926723	1.86 (1.46-2.37)	5.2E-07	6.9	13.5	0.33	1.83 (1.43-2.33)	1.1E-06	8.0	25.2	0.24
rs1926722	1.86 (1.46-2.37)	5.0E-07	6.9	13.6	0.33	1.83 (1.44-2.33)	1.1E-06	8.0	25.2	0.24
rs11122575	1.86 (1.46-2.37)	4.8E-07	7.0	13.9	0.32	1.83 (1.44-2.34)	1.0E-06	8.0	25.5	0.23
rs11568056	1.84 (1.45-2.34)	7.1E-07	7.8	23.6	0.25	1.82 (1.42-2.31)	1.4E-06	9.0	33.2	0.17
rs11122576	1.76 (1.39-2.23)	3.0E-06	7.9	24.0	0.25	1.74 (1.37-2.21)	5.4E-06	9.0	33.0	0.18
rs11568028	1.76 (1.39-2.23)	3.0E-06	7.9	23.9	0.25	1.74 (1.37-2.21)	5.4E-06	8.9	32.8	0.18
rs11122578	1.76 (1.39-2.24)	2.9E-06	7.9	24.3	0.24	1.74 (1.37-2.21)	5.3E-06	9.0	33.1	0.18
rs3789679	1.78 (1.40-2.26)	3.0E-06	7.1	15.3	0.31	1.75 (1.37-2.23)	5.9E-06	8.1	25.8	0.23
rs9804147	1.65 (1.32-2.05)	1.1E-05	6.4	6.6	0.38	1.63 (1.31-2.04)	1.7E-05	6.7	10.4	0.35
rs9804153	1.64 (1.31-2.05)	1.2E-05	6.4	6.1	0.38	1.62 (1.30-2.03)	1.8E-05	6.6	9.6	0.36
rs4028824	1.64 (1.32-2.05)	1.1E-05	6.2	3.8	0.40	1.63 (1.30-2.03)	1.7E-05	6.5	7.1	0.37
rs10864773	1.68 (1.33-2.12)	1.1E-05	6.1	1.9	0.41	1.67 (1.32-2.11)	1.8E-05	6.9	13.1	0.33
rs61762467	1.90 (1.50-2.41)	1.3E-07	8.0	25.1	0.24	1.87 (1.47-2.38)	2.8E-07	9.2	34.4	0.17
Signal 2										
rs4846857	0.66 (0.53-0.82)	1.8E-04	21.6	72.2	0.00	0.65 (0.52-0.81)	1.4E-04	22.8	73.7	0.00
rs79434380	4.02 (2.14-7.54)	1.5E-05	5.5	0.0	0.48	4.00 (2.12-7.54)	1.8E-05	5.0	0.0	0.55
rs564325629	4.29 (2.29-8.06)	5.8E-06	6.0	0.0	0.43	4.29 (2.27-8.08)	6.9E-06	6.3	4.4	0.39
rs147121532	4.43 (2.35-8.33)	3.9E-06	6.1	1.1	0.42	4.42 (2.34-8.34)	4.7E-06	6.4	5.7	0.38
rs28605378	1.61 (1.23-2.10)	4.9E-04	4.3	0.0	0.64	1.60 (1.22-2.09)	6.1E-04	4.3	0.0	0.64
rs12095859	1.63 (1.24-2.12)	3.6E-04	4.2	0.0	0.65	1.62 (1.24-2.11)	4.5E-04	4.2	0.0	0.65
rs12091328	1.62 (1.24-2.11)	3.4E-04	4.5	0.0	0.61	1.61 (1.23-2.10)	4.4E-04	4.5	0.0	0.61
rs12059171	1.64 (1.26-2.13)	2.5E-04	4.5	0.0	0.61	1.63 (1.25-2.12)	3.2E-04	4.5	0.0	0.60

rs12060898	1.65 (1.27-2.14)	1.8E-04	4.8	0.0	0.58	1.64 (1.26-2.13)	2.4E-04	4.8	0.0	0.58
rs75991123	1.72 (1.30-2.28)	1.3E-04	7.3	18.1	0.29	1.69 (1.28-2.24)	2.4E-04	7.2	17.1	0.30
chr5:156903410-157903410, associated with rectal bleeding										
rs17055178	1.88 (1.52-2.33)	4.9E-09	3.4	0.0	0.64	--	--	--	--	--
rs13180537	1.91 (1.51-2.43)	8.1E-08	5.3	6.0	0.38	--	--	--	--	--
rs78394554	1.85 (1.49-2.30)	2.7E-08	4.1	0.0	0.53	--	--	--	--	--
rs34395161	1.89 (1.49-2.40)	1.4E-07	5.3	6.0	0.38	--	--	--	--	--
rs35327501	1.82 (1.47-2.26)	5.9E-08	3.9	0.0	0.56	--	--	--	--	--
rs4704767	1.68 (1.38-2.04)	2.4E-07	3.1	0.0	0.69	--	--	--	--	--
rs35929592	1.68 (1.38-2.04)	2.4E-07	3.0	0.0	0.69	--	--	--	--	--
rs35766682	1.66 (1.37-2.03)	4.2E-07	3.4	0.0	0.64	--	--	--	--	--
rs10515757	1.68 (1.38-2.04)	2.4E-07	3.1	0.0	0.69	--	--	--	--	--
rs35153425	1.68 (1.38-2.05)	2.3E-07	3.1	0.0	0.68	--	--	--	--	--
rs17055241	1.68 (1.38-2.05)	2.3E-07	3.1	0.0	0.68	--	--	--	--	--
rs1040926	1.68 (1.38-2.05)	2.4E-07	3.1	0.0	0.68	--	--	--	--	--
rs13179825	1.68 (1.38-2.05)	2.2E-07	3.1	0.0	0.68	--	--	--	--	--
rs13184115	1.67 (1.37-2.03)	3.5E-07	3.2	0.0	0.67	--	--	--	--	--
rs17229231	1.67 (1.37-2.03)	3.1E-07	2.9	0.0	0.72	--	--	--	--	--
chr9:30366808-31366808, associated with decreased urinary stream										
rs10969913	4.59 (2.76-7.63)	4.1E-09	11.3	55.6	0.05	--	--	--	--	--
rs7868409	4.49 (2.75-7.35)	2.2E-09	8.6	42.0	0.13	--	--	--	--	--
rs10969915	4.55 (2.74-7.54)	4.5E-09	9.9	49.3	0.08	--	--	--	--	--
rs10969916	4.53 (2.72-7.54)	6.3E-09	9.8	48.9	0.08	--	--	--	--	--
rs1412406	4.55 (2.74-7.54)	4.5E-09	9.9	49.4	0.08	--	--	--	--	--
rs539024322	4.13 (2.50-6.79)	2.6E-08	10.3	51.2	0.07	--	--	--	--	--
rs10969918	4.73 (2.90-7.74)	5.5E-10	8.7	42.8	0.12	--	--	--	--	--
rs112134389	4.68 (2.84-7.72)	1.5E-09	8.7	42.3	0.12	--	--	--	--	--
rs111692482	4.68 (2.84-7.72)	1.4E-09	8.6	42.1	0.12	--	--	--	--	--
rs10969920	4.74 (2.90-7.74)	5.1E-10	8.7	42.8	0.12	--	--	--	--	--
rs10969923	4.50 (2.72-7.46)	5.2E-09	10.5	52.4	0.06	--	--	--	--	--
rs73644367	4.66 (2.81-7.74)	2.5E-09	8.5	52.8	0.08	--	--	--	--	--
rs73644368	4.64 (2.80-7.69)	2.8E-09	8.9	44.0	0.11	--	--	--	--	--
rs10969926	4.74 (2.91-7.75)	4.9E-10	8.5	52.7	0.08	--	--	--	--	--

* Single variant summary statistics. Abbreviations: HR, hazard ratio; CI, confidence interval; -- indicates that a value was not available.

† Two-sided P_{meta} was calculated using a Wald test.

‡ Two-sided P_{meta} was calculated using a chi-square test.

Supplementary Table 6. ENCODE regulatory regions overlapping with credible causal variants.

rsid	Encode ID	Regulatory region_Tissue
chr1:230337180-231337180, associated with hematuria		
Signal 1		
rs11122572	ENCFF940MJZ	Enhancer_caudate nucleus
rs4846866	ENCFF940MJZ	Enhancer_caudate nucleus
rs61762468	ENCFF940MJZ	Enhancer_caudate nucleus
rs56117713	ENCFF940MJZ	Enhancer_caudate nucleus
rs16852352	ENCFF131AMT, ENCFF345YBN, ENCFF458GST, ENCFF940MJZ	Enhancer_HepG2, Enhancer_HepG2, Enhancer_HepG2, Enhancer_caudate nucleus
rs11122573	ENCFF131AMT, ENCFF345YBN, ENCFF458GST, ENCFF940MJZ	Enhancer_HepG2, Enhancer_HepG2, Enhancer_HepG2, Enhancer_caudate nucleus
rs10864770	ENCFF131AMT, ENCFF345YBN, ENCFF458GST, ENCFF940MJZ	Enhancer_HepG2, Enhancer_HepG2, Enhancer_HepG2, Enhancer_caudate nucleus
rs10864771	ENCFF131AMT, ENCFF345YBN, ENCFF458GST, ENCFF940MJZ	Enhancer_HepG2, Enhancer_HepG2, Enhancer_HepG2, Enhancer_caudate nucleus
rs11122574	ENCFF458GST	Enhancer_HepG2
rs1926723	ENCFF458GST	Enhancer_HepG2
rs1926722	ENCFF458GST	Enhancer_HepG2
rs11122575	ENCFF131AMT, ENCFF345YBN, ENCFF357FRW, ENCFF458GST, ENCFF768HKR, ENCFF940MJZ	Enhancer_HepG2, Enhancer_HepG2, Enhancer_layer of hippocampus, Enhancer_HepG2, Promoter_HepG2, Enhancer_caudate nucleus
rs11568056	ENCFF458GST, ENCFF529FRB, ENCFF603HRN, ENCFF768HKR, ENCFF856JWA, ENCFF925ARX, ENCFF940MJZ	Enhancer_HepG2, Promoter_caudate nucleus, Promoter_liver, Promoter_HepG2, Enhancer_liver, Promoter_HepG2, Enhancer_caudate nucleus
rs11122576	ENCFF021ERU, ENCFF131AMT, ENCFF345YBN, ENCFF458GST, ENCFF529FRB, ENCFF603HRN, ENCFF662TKM, ENCFF768HKR, ENCFF856JWA, ENCFF925ARX, ENCFF940MJZ	Enhancer_HepG2, Enhancer_middle frontal area 46, Enhancer_HepG2, Enhancer_HepG2, Promoter_caudate nucleus, Promoter_liver,Promoter_heart left ventricle, Promoter_HepG2, Enhancer_liver, Promoter_HepG2, Enhancer_caudate nucleus
rs11568028	ENCFF021ERU, ENCFF131AMT, ENCFF345YBN, ENCFF357FRW, ENCFF458GST, ENCFF529FRB, ENCFF603HRN, ENCFF662TKM, ENCFF768HKR, ENCFF856JWA, ENCFF925ARX, ENCFF940MJZ	Enhancer_HepG2, Enhancer_middle frontal area 46, Enhancer_HepG2, Enhancer_layer of hippocampus, Enhancer_HepG2, Promoter_caudate nucleus, Promoter_liver,Promoter_heart left ventricle, Promoter_HepG2,Enhancer_liver, Promoter_HepG2, Enhancer_caudate nucleus
rs11122578	ENCFF021ERU, ENCFF026HMJ, ENCFF084LBA, ENCFF131AMT, ENCFF138BOV, ENCFF144PJO, ENCFF154VRY, ENCFF169ILY, ENCFF232DYU, ENCFF283LOL, ENCFF305UOC, ENCFF345YBN, ENCFF357FRW, ENCFF407NSM, ENCFF458GST, ENCFF468LNN, ENCFF518VHQ, ENCFF524SKZ, ENCFF529FRB, ENCFF602NDV, ENCFF603HRN, ENCFF608IMQ, ENCFF621WVX, ENCFF662TKM, ENCFF663YMC, ENCFF690YPD, ENCFF699XIX, ENCFF768HKR, ENCFF798VKK, ENCFF809ZVL, ENCFF826LIL, ENCFF856JWA, ENCFF920NZB, ENCFF925ARX, ENCFF937LPQ, ENCFF940MJZ, ENCFF952DET, ENCFF955CNN, ENCFF978UAS	Enhancer_HepG2, Enhancer_large intestine, Enhancer_middle frontal area 46, Enhancer_myotube, Enhancer_substantia nigra,Enhancer_small intestine, Enhancer_skeletal muscle tissue, Enhancer_pancreas, Enhancer_small intestine, Enhancer_temporal lobe, Promoter_skeletal muscle tissue, Enhancer_IMR-90, Enhancer_HepG2, Enhancer_layer of hippocampus, Promoter_large intestine, Enhancer_HepG2, Enhancer_muscle of trunk, Enhancer_cingulate gyrus, Enhancer_heart left ventricle,Promoter_caudate nucleus, Enhancer_psoas muscle, Promoter_liver, Promoter_skeletal muscle myoblast, Enhancer_angular gyrus, Promoter_heart left ventricle, Enhancer_muscle of trunk, Enhancer_psoas muscle, Enhancer_myotube, Promoter_HepG2, Enhancer_right cardiac atrium, Enhancer_heart right ventricle, Promoter_myotube,Enhancer_liver, Enhancer_muscle of leg, Promoter_HepG2,Enhancer_large intestine, Enhancer_caudate nucleus, Enhancer_adrenal gland, Enhancer_muscle of leg
rs3789679	ENCFF212AGQ	Enhancer_fibroblast of lung
rs9804147	--	--

rs9804153	--	--
rs4028824	--	--
rs10864773	ENCFF940MJZ	Enhancer_caudate nucleus
rs61762467	ENCFF033LLU, ENCFF154VRY,ENCFF447BWN, ENCFF875CFO, ENCFF937LPQ, ENCFF978UAS	Enhancer_large intestine, Enhancer_mucosa of rectum, Enhancer_pancreas,Enhancer_B cell, Enhancer_B cell, Enhancer_large intestine
Signal 2		
rs4846857	ENCFF518VHQ	Enhancer_cingulate gyrus
rs79434380	--	--
rs564325629	--	--
rs147121532	ENCFF043YPD	Enhancer_mucosa of rectum
rs28605378	ENCFF043YPD, ENCFF715DPV, ENCFF892DRC	Enhancer_mucosa of rectum,Enhancer_stomach,Enhancer_stomach
rs12095859	ENCFF013DHK, ENCFF033LLU, ENCFF043YPD, ENCFF084LBA, ENCFF183FMS, ENCFF412OXP, ENCFF519JQV, ENCFF605WXQ, ENCFF715DPV, ENCFF741KDK, ENCFF892DRC	Enhancer_MCF-7, Enhancer_mucosa of rectum, Enhancer_mucosa of rectum, Enhancer_substantia nigra, Enhancer_stomach, Promoter_stomach, Enhancer_colonic mucosa, Enhancer_stomach, Enhancer_stomach, Enhancer_sigmoid colon, Enhancer_stomach
rs12091328	ENCFF013DHK, ENCFF021ERU, ENCFF033LLU, ENCFF043YPD, ENCFF084LBA, ENCFF357FRW, ENCFF518VHQ, ENCFF621WVX, ENCFF715DPV, ENCFF741KDK, ENCFF892DRC	Enhancer_MCF-7,Enhancer_middle frontal area 46 ,Enhancer_mucosa of rectum,Enhancer_mucosa of rectum,Enhancer_substantia nigra,Enhancer_layer of hippocampus,Enhancer_cingulate gyrus,Enhancer_angular gyrus,Enhancer_stomach,Enhancer_sigmoid colon,Enhancer_stomach
rs12059171	ENCFF013DHK,ENCFF021ERU,ENCFF033LLU,ENCFF043YPD,ENCFF059PMT,ENCFF084LBA,ENCFF131AMT,ENCFF138BOV,ENCFF183FMS,ENCFF232DYU,ENCFF345YBN,ENCFF357FRW,ENCFF458GST,ENCFF518VHQ,ENCFF519JQV,ENCFF605WXQ,ENCFF621WVX,ENCFF715DPV,ENCFF741KDK,ENCFF892DRC,ENCFF937LPQ,ENCFF940MJZ	Enhancer_HepG2,Enhancer_MCF-7,Enhancer_middle frontal area 46 ,Enhancer_mucosa of rectum,Enhancer_mucosa of rectum,Enhancer_lung,Enhancer_substantia nigra,Enhancer_small intestine,Enhancer_stomach,Enhancer_temporal lobe,Enhancer_HepG2,Enhancer_layer of hippocampus,Enhancer_HepG2,Enhancer_cingulate gyrus,Enhancer_colonic mucosa,Enhancer_stomach,Enhancer_angular gyrus,Enhancer_stomach,Enhancer_sigmoid colon,Enhancer_stomach,Enhancer_large intestine,Enhancer_caudate nucleus
rs12060898	--	--
rs75991123	--	--
chr5:156903410-157903410, associated with rectal bleeding		
rs17055178	--	--
rs13180537	--	--
rs78394554	ENCFF031NTY,ENCFF242UFI	Enhancer_mesendoderm, Enhancer_mesendoderm
rs34395161	ENCFF031NTY,ENCFF242UFI	Enhancer_mesendoderm, Enhancer_mesendoderm
rs35327501	ENCFF937LPQ,ENCFF952DET,ENCFF953YED,ENCFF978UAS	Enhancer_large intestine, Enhancer_large intestine, Enhancer_adrenal gland, Enhancer_adrenal gland
rs4704767	--	--
rs35929592	--	--
rs35766682	ENCFF446JOJ,ENCFF952DET,ENCFF953YED	Enhancer_esophagus, Enhancer_adrenal gland, Enhancer_adrenal gland
rs10515757	ENCFF952DET,ENCFF953YED	Enhancer_adrenal gland, Enhancer_adrenal gland
rs35153425	--	--
rs17055241	--	--
rs1040926	ENCFF210ALH,ENCFF595MLU,ENCFF733LQH	Enhancer_keratinocyte, Enhancer_foreskin keratinocyte, Enhancer_keratinocyte

rs13179825	ENCFF210ALH,ENCFF595MLU,ENCFF733LQH	Enhancer_keratinocyte, Enhancer_foreskin keratinocyte, Enhancer_keratinocyte
rs13184115	ENCFF210ALH,ENCFF733LQH	Enhancer_keratinocyte, Enhancer_keratinocyte
rs17229231	--	--

Abbreviations: -- indicates that a value was not available.

Supplementary Table 7. Top-ranking genes ($p < 0.001$) associated with each toxicity outcome.

Toxicity by Location	Gene ID	Gene symbol	Gene Type	No. of SNPs	p-value*
Rectal bleeding					
chr16	100130958	*SYCE1L*	protein coding	369	3.22x10-4
chr16	22879	*MON1B*	protein coding	330	3.40x10-4
chr3	55096	*EBLN2*	protein coding	261	5.45x10-4
chr4	100874374	*ARHGEF38-IT1*	protein coding	188	5.72x10-4
chr2	5498	*KIDINS220*	protein coding	251	6.38x10-4
chr4	54848	*ARHGEF38*	protein coding	244	6.83x10-4
chr5	2559	*GABRA6*	protein coding	248	7.55x10-4
chr2	151254	*ALS2CR11*	protein coding	274	7.94x10-4
chr6	168002	*DACT2*	protein coding	439	8.27x10-4
chr3	151987	*PPP4R2*	protein coding	482	8.30x10-4
chr7	79571	*GCC1*	protein coding	126	9.90x10-4
Increased urinary frequency					
chr14	145567	*TTC7B*	ncRNA	1031	9.37x10-5
chr14	9623	*TCL1B*	protein coding	418	1.27x10-4
chr20	63935	*PCIF1*	protein coding	162	1.86x10-4
chr15	9836	*LCMT2*	protein coding	154	3.16x10-4
chr14	27004	*TCL6*	ncRNA	465	3.24x10-4
chr20	5360	*PLTP*	protein coding	193	3.50x10-4
chr15	161823	*ADAL*	protein coding	179	3.73x10-4
chr12	8738	*CRADD*	protein coding	34	4.56x10-4
chr15	116179	*TGM7*	protein coding	166	4.75x10-4
chr19	284382	*ACTL9*	protein coding	229	5.58x10-4
chr15	146050	*ZSCAN29*	protein coding	169	6.46x10-4
chr22	266697	*POM121L4P*	pseudo	228	6.51x10-4
chr13	196541	*METTL21C*	protein coding	309	6.91x10-4
chr20	5476	*CTSA*	protein coding	178	7.05x10-4
chr22	84222	*TMEM191A*	pseudo	222	7.24x10-4
chr22	5297	*PI4KA*	protein coding	500	7.90x10-4
chr22	3053	*SERPIND1*	protein coding	228	8.42x10-4
chr15	27229	*TUBGCP4*	protein coding	214	9.36x10-4
chr17	100505576	*LINC00672*	ncRNA	175	9.42x10-4
chr4	6452	*SH3BP2*	protein coding	274	9.76x10-4
chr20	140825	*NEURL2*	protein coding	168	9.92x10-4
chr12	225	*ABCD2*	protein coding	6	9.99x10-4
Decreased urinary stream					
chr16	54925	*ZSCAN32*	protein coding	360	1.14x10-5
chr16	7727	*ZNF174*	protein coding	307	1.20x10-5
chr14	26257	*NKX2-8*	protein coding	253	1.24x10-5
chr16	100463285	*MTRNR2L4*	protein coding	292	2.00x10-5
chr16	4993	*OR2C1*	ncRNA	286	2.29x10-5
chr16	79903	*NAA60*	protein coding	357	6.89x10-5
chr16	146434	*ZNF597*	pseudo	337	6.95x10-5
chr16	7627	*ZNF75A*	protein coding	243	7.06x10-5
chr9	100616351	*MIR4670*	protein coding	139	8.25x10-5
chr15	8924	*HERC2*	protein coding	104	8.41x10-5

chr9	2649	*NR6A1*	protein coding	170	8.72x10-5
chr9	1842	*ECM2*	protein coding	186	9.69x10-5
chr9	2516	*NR5A1*	protein coding	125	1.19x10-4
chr14	4140	*MARK3*	protein coding	471	1.24x10-4
chr14	7080	*NKX2-1*	protein coding	164	1.28x10-4
chr9	347088	*GPR144*	protein coding	149	1.59x10-4
chr11	100616311	*MIR3973*	ncRNA	157	1.65x10-4
chr14	5083	*PAX9*	protein coding	268	1.67x10-4
chr2	3635	*INPP5D*	protein coding	689	1.76x10-4
chr14	253970	*SFTA3*	protein coding	220	1.89x10-4
chr9	54829	*ASPN*	protein coding	138	2.54x10-4
chr9	401541	*CENPP*	protein coding	389	2.68x10-4
chr9	100379345	*MIR181A2HG*	ncRNA	48	2.76x10-4
chr9	406954	*MIR181A2*	ncRNA	39	3.15x10-4
chr9	406956	*MIR181B2*	ncRNA	39	3.19x10-4
chr9	23511	*NUP188*	protein coding	117	4.64x10-4
chr9	56904	*SH3GLB2*	protein coding	70	4.71x10-4
chr14	1152	*CKB*	protein coding	210	4.76x10-4
chr9	22845	*DOLK*	protein coding	78	5.84x10-4
chr9	4958	*OMD*	protein coding	110	5.88x10-4
chr19	728752	*LOC728752*	ncRNA	230	6.31x10-4
chr9	254295	*PHYHD1*	protein coding	90	6.47x10-4
chr9	169611	*OLFML2A*	protein coding	161	6.90x10-4
chr3	401097	*C3orf80*	protein coding	105	7.15x10-4
chr3	255758	*TCTEX1D2*	protein coding	300	8.92x10-4
chr18	54808	*DYM*	protein coding	973	9.18x10-4
chr9	4969	*OGN*	protein coding	115	9.29x10-4
chr9	56262	*LRRC8A*	protein coding	106	9.49x10-4
Hematuria					
chr6	94120	*SYTL3*	protein coding	595	2.82x10-5
chr6	101409257	*EZR-AS1*	ncRNA	309	5.86x10-5
chr1	183	*AGT*	protein coding	370	7.91x10-5
chr6	202459	*OSTCP1*	pseudo	301	8.65x10-5
chr6	7430	*EZR*	protein coding	463	9.48x10-5
chr4	80144	*FRAS1*	protein coding	1570	1.19x10-4
chr6	100500851	*MIR3918*	ncRNA	333	1.38x10-4
chr20	650	*BMP2*	protein coding	227	2.76x10-4
chr1	22796	*COG2*	protein coding	425	2.98x10-4
chr11	63901	*FAM111A*	protein coding	106	3.58x10-4
chr5	23037	*PDZD2*	protein coding	1522	3.61x10-4
chr15	283726	*FAM154B*	protein coding	145	4.05x10-4
chr7	100288524	*LOC100288524*	ncRNA	486	4.38x10-4
chr10	55130	*ARMC4*	protein coding	492	5.27x10-4
chr11	374393	*FAM111B*	protein coding	131	5.54x10-4
chr1	10753	*CAPN9*	protein coding	486	6.46x10-4
chr15	390660	*LOC390660*	pseudo	94	6.86x10-4
chr8	286097	*MICU3*	protein coding	463	7.01x10-4
chr17	6426	*SRSF1*	protein coding	91	7.34x10-4
chr17	1277	*COL1A1*	protein coding	204	7.36x10-4
chr15	79631	*EFTUD1*	protein coding	413	7.38x10-4
chr11	23220	*DTX4*	protein coding	174	7.99x10-4

chr12	6579	*SLCO1A2*	protein coding	31	8.53x10-4
chr8	100874052	*LINC00534*	ncRNA	509	8.65x10-4
chr15	161742	*SPRED1*	protein coding	557	9.56x10-4
chr12	10599	*SLCO1B1*	protein coding	26	9.80x10-4

***** Two-sided P_{meta} was calculated using a Wald test**.**

Supplementary Table 8. Pathway scores for top-ranking pathways (chi-square p < 0.05) associated with each toxicity outcome.

Toxicity	Pathway Name	chi2 pvalue*	empirical pvalue†
Rectal bleeding	REACTOME_PEROXISOMAL_LIPID_METABOLISM	6.04x10-4	6.70x10-4
	BIOCARTA_PYK2_PATHWAY	0.006	0.005
	REACTOME_GABA_RECEPTOR_ACTIVATION	0.009	0.007
	REACTOME_TRNA_AMINOACYLATION	0.01	0.01
	KEGG_PEROXISOME	0.01	0.01
	BIOCARTA_GPCR_PATHWAY	0.01	0.01
	REACTOME_INWARDLY_RECTIFYING_K_CHANNELS	0.01	0.009
	BIOCARTA_INTEGRIN_PATHWAY	0.01	0.01
	BIOCARTA_CHEMICAL_PATHWAY	0.01	0.01
	BIOCARTA_FMLP_PATHWAY	0.01	0.01
	REACTOME_TRANSCRIPTIONAL_REGULATION_OF_WHITE_ADIPOCYTE_DIFFERENTIATION*	0.01	0.02
	REACTOME_CA_DEPENDENT_EVENTS	0.01	0.01
	BIOCARTA_AT1R_PATHWAY	0.01	0.01
	BIOCARTA_BCR_PATHWAY	0.02	0.01
	REACTOME_A_TETRASACCHARIDE_LINKER_SEQUENCE_IS_REQUIRED_FOR_GAG_SYNTHESIS	0.02	0.01
	BIOCARTA_UCALPAIN_PATHWAY†	0.02	0.02
	KEGG_GLYCOSPHINGOLIPID_BIOSYNTHESIS_GLOBO_SERIES	0.02	0.02
	REACTOME_OPIOID_SIGNALLING	0.02	0.02
	BIOCARTA_CBL_PATHWAY	0.02	0.02
	BIOCARTA_TNFR1_PATHWAY	0.02	0.02
	BIOCARTA_FCER1_PATHWAY	0.02	0.02
	BIOCARTA_ECM_PATHWAY‡	0.03	0.03
	REACTOME_CIRCADIAN_REPRESSION_OF_EXPRESSION_BY_REV_ERBA	0.03	0.03
	REACTOME_ARMS_MEDIATED_ACTIVATION	0.03	0.04
	REACTOME_CYTOSOLIC_TRNA_AMINOACYLATION	0.03	0.03
	REACTOME_MEIOTIC_RECOMBINATION	0.03	0.03
	BIOCARTA_FAS_PATHWAY	0.03	0.03
	REACTOME_GABA_B_RECEPTOR_ACTIVATION	0.03	0.03
	BIOCARTA_IL3_PATHWAY	0.03	0.03
	KEGG_TYPE_II_DIABETES_MELLITUS†	0.03	0.03
	BIOCARTA_TCR_PATHWAY	0.03	0.03
	REACTOME_PLC_BETA_MEDIATED_EVENTS	0.03	0.03
	REACTOME_GLYCOLYSIS	0.03	0.03
	KEGG_NATURAL_KILLER_CELL_MEDIATED_CYTOTOXICITY	0.03	0.03
	BIOCARTA_AGR_PATHWAY	0.03	0.03
	BIOCARTA_NUCLEARRS_PATHWAY	0.04	0.03
	REACTOME_PROSTACYCLIN_SIGNALLING_THROUGH_PROSTACYCLIN_RECEPTOR	0.04	0.03
	KEGG_CARDIAC_MUSCLE_CONTRACTION	0.04	0.04
	KEGG_AMINOACYL_TRNA_BIOSYNTHESIS	0.04	0.04

Rectal bleeding	REACTOME_RORA_ACTIVATES_CIRCADIAN_EXPRESSION	0.04	0.05
	BIOCARTA_MCALPAIN_PATHWAY	0.04	0.04
	REACTOME_DAG_AND_IP3_SIGNALING	0.04	0.04
	KEGG_P53_SIGNALING_PATHWAY	0.04	0.04
	BIOCARTA_NDKDYNAMIN_PATHWAY[‡]	0.04	0.04
	REACTOME_ERKS_ARE_INACTIVATED	0.04	0.04
	REACTOME_FACILITATIVE_NA_INDEPENDENT_GLUCOSE_TRANSPORTERS	0.04	0.04
	BIOCARTA_NOS1_PATHWAY	0.04	0.04
	BIOCARTA_RACCYCD_PATHWAY	0.04	0.04
	BIOCARTA_GABA_PATHWAY	0.04	0.05
	REACTOME_INHIBITION_OF_VOLTAGE_GATED_CA2_CHANNELS_VIA_GBETA_GAMMA_SUBUNITS	0.04	0.04
	BIOCARTA_THELPER_PATHWAY	0.04	0.05
	BIOCARTA_DREAM_PATHWAY	0.05	0.04
	BIOCARTA_CERAMIDE_PATHWAY	0.05	0.05
	BIOCARTA_FREE_PATHWAY	0.05	0.04
	REACTOME_GLUCAGON_SIGNALING_IN_METABOLIC_REGULATION	0.05	0.05
	BIOCARTA_EGFR_SMRTE_PATHWAY	0.05	0.05
	REACTOME_BINDING_AND_ENTRY_OF_HIV_VIRION	0.05	0.05
	REACTOME_MITOCHONDRIAL_TRNA_AMINOACYLATION	0.05	0.05
	BIOCARTA_NFAT_PATHWAY[†]	0.05	0.05
Increased urinary frequency	REACTOME_PURINE_METABOLISM	0.001	0.001
	REACTOME_BASIGIN_INTERACTIONS[§]	0.005	0.004
	REACTOME_PURINE_CATABOLISM	0.006	0.005
	REACTOME_APOPTOSIS_INDUCED_DNA_FRAGMENTATION	0.009	0.009
	KEGG_SELENOAMINO_ACID_METABOLISM	0.01	0.01
	REACTOME_VOLTAGE_GATED_POTASSIUM_CHANNELS	0.01	0.01
	REACTOME_STRIATED_MUSCLE_CONTRACTION	0.02	0.01
	REACTOME_ABACAVIR_TRANSPORT_AND_METABOLISM	0.02	0.02
	REACTOME_TRANSCRIPTIONAL_REGULATION_OF_WHITE_ADIPOCYTE_DIFFERENTIATION[*]	0.02	0.02
	REACTOME_PURINE_SALVAGE	0.02	0.02
	REACTOME_CELL_SURFACE_INTERACTIONS_AT_THE_VASCULAR_WALL	0.02	0.02
	REACTOME_ETHANOL_OXIDATION	0.02	0.02
	REACTOME_CD28_DEPENDENT_VAV1_PATHWAY	0.02	0.02
	REACTOME_ALPHA_LINOLENIC_ACID_ALA_METABOLISM	0.02	0.03
	KEGG_PURINE_METABOLISM	0.03	0.02
	KEGG_INOSITOL_PHOSPHATE_METABOLISM	0.03	0.03
	KEGG_VIRAL_MYOCARDITIS	0.03	0.03
Increased urinary frequency	REACTOME_N_GLYCAN_TRIMMING_IN_THE_ER_AND_CALNEXIN_CALRETICULIN_CYCLE	0.03	0.03
	REACTOME_SIGNAL_TRANSDUCTION_BY_L1	0.03	0.03
	KEGG_RETINOL_METABOLISM	0.03	0.03
	REACTOME_CALNEXIN_CALRETICULIN_CYCLE	0.04	0.04
	REACTOME_HDL_MEDIATED_LIPID_TRANSPORT	0.04	0.04
	REACTOME_CS_DS_DEGRADATION	0.04	0.03

	BIOCARTA_DNAFRAGMENT_PATHWAY	0.04	0.04
	BIOCARTA_VDR_PATHWAY	0.04	0.04
	REACTOME_COPI_MEDIATED_TRANSPORT	0.04	0.04
	BIOCARTA_CDK5_PATHWAY	0.04	0.05
	KEGG_RENIN_ANGIOTENSIN_SYSTEM	0.05	0.05
Decreased urinary stream	REACTOME_CHROMOSOME_MAINTENANCE	0.005	0.005
	REACTOME_CHOLESTEROL_BIOSYNTHESIS	0.006	0.009
	REACTOME_CASPASE_MEDIATED_CLEAVAGE_OF_CYTOSKELETAL_PROTEINS	0.01	0.01
	REACTOME_MITOTIC_PROMETAPHASE	0.01	0.009
	REACTOME_MEIOTIC_SYNAPSIS	0.01	0.02
	REACTOME_XENOBIOTICS	0.01	0.02
	BIOCARTA_CDMAC_PATHWAY	0.01	0.02
	REACTOME_MITOTIC_M_M_G1_PHASES	0.02	0.01
	BIOCARTA_BIOPEPTIDES_PATHWAY[‖]	0.02	0.02
	REACTOME_CELL_CYCLE	0.02	0.02
	REACTOME_DNA_REPLICATION	0.02	0.02
	REACTOME_DEPOSITION_OF_NEW_CENPA_CONTAINING_NUCLEOSOMES_AT_THE_CENTROMERE	0.02	0.01
	REACTOME_OLFACTORY_SIGNALING_PATHWAY	0.02	0.01
	REACTOME_CELL_CYCLE_MITOTIC	0.03	0.03
	REACTOME_RAF_MAP_KINASE_CASCADE	0.03	0.04
	REACTOME_SYNTHESIS_OF_PC	0.03	0.03
	BIOCARTA_ECM_PATHWAY[‡]	0.03	0.04
	KEGG_FC_EPSILON_RI_SIGNALING_PATHWAY	0.04	0.03
	KEGG_FC_GAMMA_R_MEDIATED_PHAGOCYTOSIS	0.04	0.03
	REACTOME_CYTOKINE_SIGNALING_IN_IMMUNE_SYSTEM[‖]	0.04	0.04
	BIOCARTA_NDKDYNAMIN_PATHWAY[‡]	0.04	0.04
	KEGG_MELANOGENESIS	0.04	0.05
	REACTOME_SHC_RELATED_EVENTS	0.04	0.05
	REACTOME_TRANSPORT_OF_RIBONUCLEOPROTEINS_INTO_THE_HOST_NUCLEUS	0.05	0.04
	REACTOME_AMINE_LIGAND_BINDING_RECEPTORS	0.05	0.05
	REACTOME_SIGNALING_BY_ILS	0.05	0.05
	BIOCARTA_RHO_PATHWAY	0.05	0.05
	REACTOME_METABOLISM_OF_RNA	0.05	0.05
Hematuria	REACTOME_PLATELET_ADHESION_TO_EXPOSED_COLLAGEN	0.002	0.002
	REACTOME_INTERFERON_ALPHA_BETA_SIGNALING	0.004	0.005
	BIOCARTA_BIOPEPTIDES_PATHWAY[‖]	0.007	0.005
	REACTOME_L1CAM_INTERACTIONS	0.008	0.005
	BIOCARTA_ALK_PATHWAY	0.008	0.008
	REACTOME_REGULATION_OF_IFNA_SIGNALING	0.009	0.009
	REACTOME_ACYL_CHAIN_REMODELLING_OF_PI	0.01	0.01
	BIOCARTA_UCALPAIN_PATHWAY[†]	0.01	0.007
	REACTOME_RECYCLING_PATHWAY_OF_L1	0.01	0.008
	BIOCARTA_ACE2_PATHWAY	0.02	0.01
	KEGG_TYPE_II_DIABETES_MELLITUS[†]	0.02	0.02

REACTOME_ACYL_CHAIN_REMODELLING_OF_PG	0.02	0.02
REACTOME_REGULATION_OF_BETA_CELL_DEVELOPMENT	0.02	0.02
REACTOME_ACTIVATION_OF_BH3_ONLY_PROTEINS	0.02	0.02
REACTOME_REGULATION_OF_IFNG_SIGNALING	0.02	0.02
KEGG_JAK_STAT_SIGNALING_PATHWAY	0.02	0.02
REACTOME_GROWTH_HORMONE_RECEPTOR_SIGNALING	0.02	0.02
REACTOME_ACTIVATED_NOTCH1_TRANSMITS_SIGNAL_TO_THE_NUCLEUS	0.03	0.02
BIOCARTA_NFAT_PATHWAY[†]	0.03	0.02
KEGG_VASCULAR_SMOOTH_MUSCLE_CONTRACTION	0.03	0.03
REACTOME_SIGNALING_BY_ACTIVATED_POINT_MUTANTS_OF_FGFR1	0.03	0.03
REACTOME_RNA_POL_I_TRANSCRIPTION	0.03	0.03
REACTOME_REGULATION_OF_GENE_EXPRESSION_IN_BETA_CELLS	0.04	0.04
REACTOME_SYNTHESIS_OF_PA	0.04	0.04
REACTOME_TRANSPORT_OF_ORGANIC_ANIONS	0.04	0.04
REACTOME_E2F_ENABLED_INHIBITION_OF_PRE_REPLICATION_COMPLEX_FORMATION	0.04	0.05
REACTOME_AXON_GUIDANCE	0.04	0.03
REACTOME_RNA_POL_I_TRANSCRIPTION_INITIATION	0.04	0.04
REACTOME_BASIGIN_INTERACTIONS[§]	0.04	0.04
BIOCARTA_CARDIACEGF_PATHWAY	0.05	0.03
REACTOME_SIGNALING_BY_BMP	0.05	0.04
BIOCARTA_PTC1_PATHWAY	0.05	0.05
REACTOME_ACYL_CHAIN_REMODELLING_OF_PS	0.05	0.05
REACTOME_INTRINSIC_PATHWAY_FOR_APOPTOSIS	0.05	0.05
REACTOME_CYTOKINE_SIGNALING_IN_IMMUNE_SYSTEM[‖]	0.05	0.05

[*] Two-sided pvalue was calculated using a chi-square test.
[†] Following methods in [23], a Monte Carlo estimate of the p-value is obtained by sampling random gene sets of size and calculating the fraction of sets reaching a higher score than gene set of the given pathway.
[‡] Pathway is associated with both rectal bleeding and increased urinary frequency
[§] Pathway is associated with both rectal bleeding and hematuria
[‖] Pathway is associated with both rectal bleeding and decreased urinary stream
[¶] Pathway is associated with both increased urinary frequency and hematuria
[#] Pathway is associated with both decreased urinary stream and hematuria

Supplementary Table 9. Evaluation of radiotoxicity risk variants in Japanese cohorts treated with either external beam photon therapy (PRRG-photon), permanent seed brachytherapy with or without external beam photon therapy (NTMC) or carbon ion therapy (PRRG-Cion).

SNP	Toxicity Outcome	Info[*]	Effect size (95% CI)		
			**In NTMC (N=254)**	**In PRRG-photon (N=170)**	**In PRRG-Cion (N=538)**
rs17055178<br>chr5:157,403,410[†]<br>MAF[‡] 0.07	Time to onset of grade 2+ rectal bleeding	0.98	NA[§]	NA[§]	NA[§]
rs10969913<br>chr9:30,866,808[b]<br>MAF[‡] 0.37	Time to onset of grade 2+ decreased urinary stream[‖]	0.98	HR = 1.15 (0.82 to 1.61)	HR = 1.83 (0.44 to 7.68)	HR = 0.90 (0.36 to 2.26)
rs11122573<br>chr1:230,837,180[†]<br>MAF[‡] 0.31	Time to onset of grade 2+ hematuria[¶]	0.99	NA[#]	NA[**]	HR = 1.18 (0.53 to 2.61)
rs147121532<br>chr1: 230,451,849[†]<br>MAF[‡] 0.09	Time to onset of grade 2+ hematuria[¶]	0.85	NA[#]	NA[**]	HR = 1.52 (0.48 to 4.84)
rs17599026<br>chr5:137,763,798[†]<br>MAF[‡] 0.11	Presence of grade 1+ increased urinary frequency at 2 years after radiotherapy[††]	0.84	OR = 1.13 (0.49 to 2.64)	OR = 1.51 (0.56 to 4.05)	OR = 0.63 (0.27 to 1.49)
rs7720298<br>chr5:13,858,328[†]<br>MAF[‡] 0.20	Presence of grade 1+ decreased urinary stream at 2 years after radiotherapy[‡‡]	0.97	OR = 1.42 (0.77 to 2.63)	NA[§§]	NA[§§]
rs1801516<br>chr11:108,175,462[†]<br>MAF[‡] 0.00	Overall toxicity	NA[‖‖]	NA[‖‖]	NA[‖‖]	NA[‖‖]
rs7582141<br>chr2:159,899,489[†]<br>MAF[‡] 0.12	Overall toxicity[¶¶]	0.99	OR = 1.07 (0.53 to 2.16)	OR = 0.97 (0.79 to 1.18)	OR = 0.95 (0.90 to 1.01)

[*] Imputation info score values are from the oncoarray. Abbreviations: SNP, single nucleotide polymorphism; CI, confidence interval; MAF, minor allele frequency; HR, hazard ratio; OR, odds ratio; NA, not analyzed.
[†] Base position according to Genome Reference Consortium Human Build 37 (hg19).
[‡] Minor allele frequency from PRACTICAL Oncoarray samples of Japanese ancestry
[§] Rectal bleeding was not assessed in NTMC or PRRG
[‖] 82 out of 262 individuals in NTMC,5 out of 170 individuals in PRRG-photon, and 11 out of 538 individuals in PRRG-Cion developed grade 2+ decreased urinary stream.
[¶] 16 out of 538 individuals in PRRG-Cion developed grade 2+ hematuria.
[#] Hematuria was not assessed in NTMC.
[**] No individuals in PRRG-photon reported grade 2 or worse toxicity and so this outcome was not analyzed.

[††] 75 out of 198 individuals in NTMC, 35 out of 98 individuals in PRRG-photon, and 49 out of 414 individuals in PRRG-Cion developed grade 1+ increased urinary frequency at 2 years after radiotherapy.
[‡‡] 58 out of 180 individuals in NTMC developed grade 1+ decreased urinary stream at 2 years after radiotherapy.
[§§] Only 2 individuals in PRRG-photon and no individuals in PRRG-Cion developed grade 1+ decreased urinary stream at 2 years after radiotherapy and so this outcome was not analyzed.
[‖‖] SNP is monomorphic in the Japanese population and was not analyzable in NTMC or PRRG.
[¶¶] Overall toxicity was measured using STAT score [26] and dichotomized by comparing those individuals with a STAT score greater than or equal to one standard deviation above the mean to individuals with a STAT score less than one standard deviation above the mean.

Supplementary Table 10. C-statistics representing model performance comparing models with only clinical factors to models including clinical factors and SNPs

Toxicity outcome	C-statistic	
	Model only clinical factors	Model with clinical factors and SNP(s)
Increased urinary frequency	0.563	0.567
Decreased urinary stream	0.561	0.575
Hematuria	0.578	0.617
Rectal bleeding	0.603	0.621

Supplementary Figures

	RAPPER-I N=658	RAPPER-II N=1,947	RADIOGEN N=677	GenePARE-I N=382	GenePARE-II N=338	UGhent N=317	CCI-BT N=258	CCI-EBRT N=155	PRRG-photon N=172	PRRG-Cion N=545	NTMC N=256
Removed: poor quality DNA	12	35	0	30	22	0	0	1	1	7	1
Removed: included in batch I	NA	339	NA	NA	4	NA	NA	NA	NA	NA	NA
Removed: Ancestry*	39	55	1	91	62	1	3	3	0	0	0
Removed: lacking covariate data†	9	6	18	5	14	5	3	3	1	0	1
Included in analysis	N=598	N=1,512	N=658	N=256	N=236	N=311	N=252	N=148	N=170	N=538	N=254

Supplementary Figure 1. Study participants. N=4,833 included in analysis (N=3,871 from European cohorts; N=962 from Japanese cohorts).

* Non-European ancestry samples were removed from RAPPER, RADIOGEN, GenePARE, UGhent, CCI-BT and CCI-EBRT. Non-Japanese ancestry samples were removed from NTMC and PRRG. Abbreviations: NA, not applicable.

† Covariates included in the GWAS meta-analysis are age, total BED, prior prostatectomy, and receipt of androgen deprivation therapy.

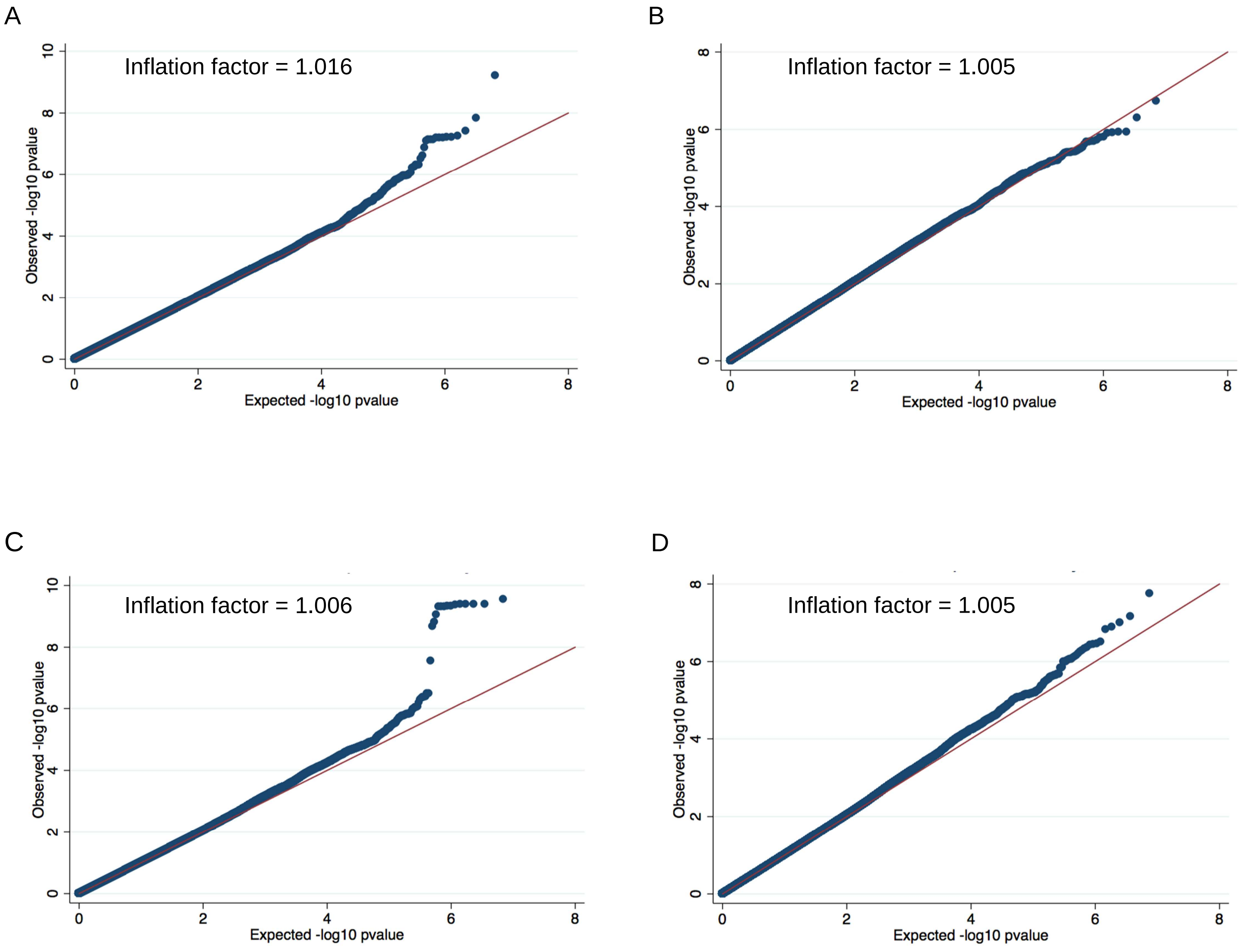

Supplementary Figure 2. QQ plots. The plots show expected and observed p-values from GWAS meta-analysis of rectal bleeding (A), increased urinary frequency (B), decreased urinary stream (C), and hematuria (D).

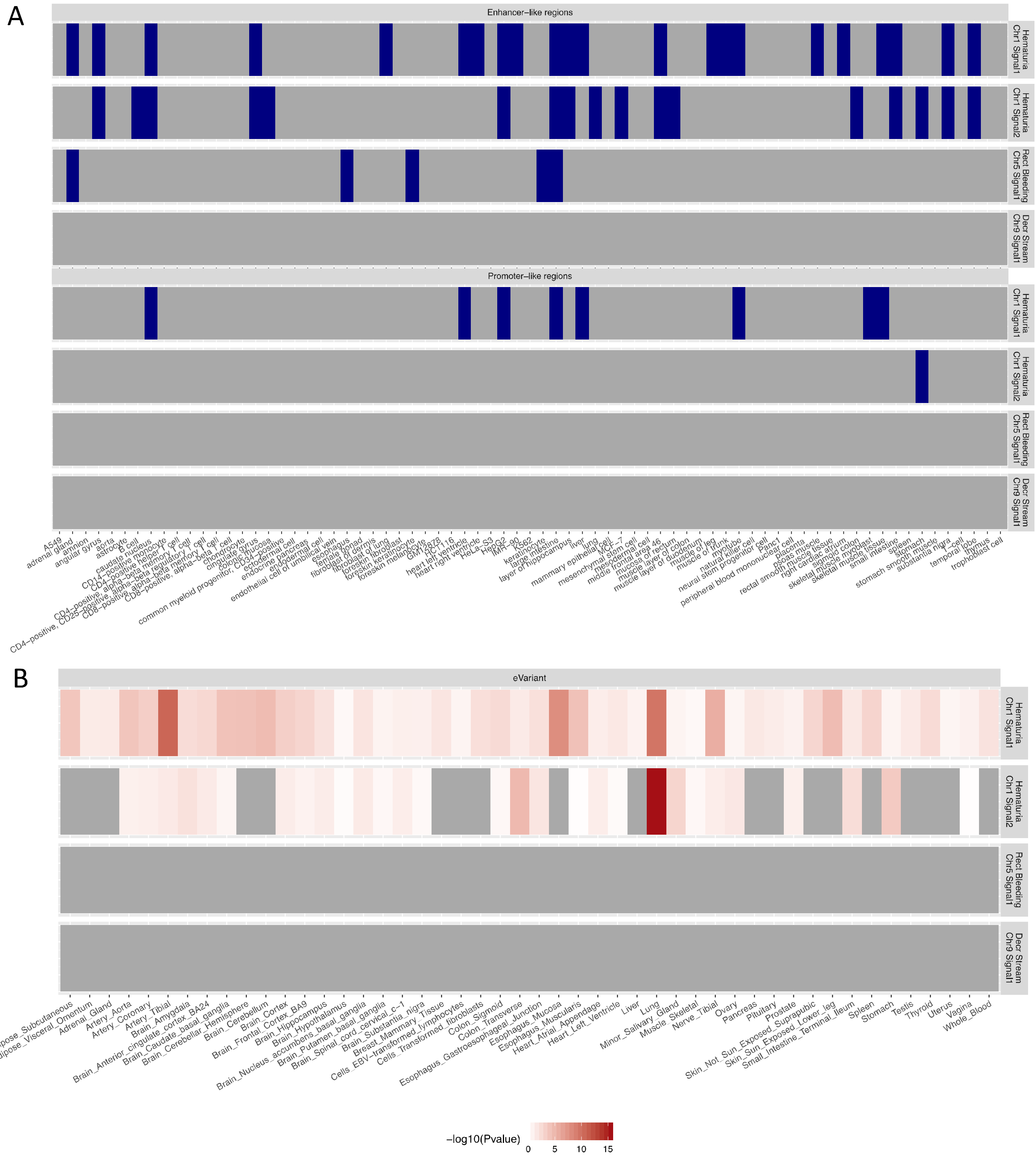

Supplementary Figure 3. Mapping of credible causal variants (CCVs). In panel A, CCVs overlap with regulatory regions, enhancer- and promoter-like according to ENCODE. X axis: cell lines or tissues. Y axis: independent signals. Top graph shows enhancer-like regions. Bottom graph shows promoter-like regions. Blue: at least one CCV overlap a regulatory region active in the specific cell-line or tissue. Dark grey: any CCV overlap an active regulatory region. Panel B shows co-localization of CCVs with variants driving the expression of a particular transcript according to GTEX. X axis: tissues. Y axis: independent signals. Red, most significant expression p-value out of all CCVs at the signal and all evaluated transcripts for that tissue. Dark grey, no significant variants driving the expression of any transcript in the evaluated tissues.
